# Discovery of key regulators of dark gland development and hypericin biosynthesis in St. John's Wort (*Hypericum perforatum*)

**DOI:** 10.1111/pbi.13141

**Published:** 2019-05-17

**Authors:** Paride Rizzo, Lothar Altschmied, Pauline Stark, Twan Rutten, André Gündel, Sarah Scharfenberg, Katrin Franke, Helmut Bäumlein, Ludger Wessjohann, Marcus Koch, Ljudmilla Borisjuk, Timothy F. Sharbel

**Affiliations:** ^1^ Leibniz‐Institut für Pflanzengenetik und Kulturpflanzenvorschung (IPK) Gatersleben Germany; ^2^ Leibniz‐Institut für Pflanzenbiochemie (IPB) Halle (Saale) Germany; ^3^ Ruprecht Karls Universität Heidelberg Heidelberg Germany; ^4^ Global Institute for Food Security (GIFS) University of Saskatchewan Saskatoon SK Canada

**Keywords:** *Hypericum perforatum*, transcriptome, metabolome, hypericin, gene discovery, dark gland

## Abstract

Hypericin is a molecule of high pharmaceutical importance that is synthesized and stored in dark glands (DGs) of St. John's Wort (*Hypericum perforatum*). Understanding which genes are involved in dark gland development and hypericin biosynthesis is important for the development of new *Hypericum* extracts that are highly demanded for medical applications. We identified two transcription factors whose expression is strictly synchronized with the differentiation of DGs. We correlated the content of hypericin, pseudohypericin, endocrocin, skyrin glycosides and several flavonoids with gene expression and DG development to obtain a revised model for hypericin biosynthesis. Here, we report for the first time genotypes which are polymorphic for the presence/total absence (G+/G−) of DGs in their placental tissues (PTs). DG development was characterized in PTs using several microscopy techniques. Fourier transform infrared microscopy was established as a novel method to precisely locate polyaromatic compounds, such as hypericin, in plant tissues. In addition, we obtained transcriptome and metabolome profiles of unprecedented resolution in *Hypericum*. This study addresses for the first time the development of dark glands and identifies genes that constitute strong building blocks for the further elucidation of hypericin synthesis, its manipulation in plants, its engineering in microbial systems and its applications in medical research.

## Introduction

The genus *Hypericum* has a worldwide distribution and includes more than 460 species occupying very diverse habitats (Crockett and Robson, 2011). The most known representative, *Hypericum perforatum*, better known as St. John's Wort, is not only a model organism for the study of apomixis (asexual reproduction) but also an ancient medicinal plant mainly used for the treatment of depressions (Galla *et al*., 2011; Rizzo, 2016; Schallau *et al*., 2010). Its secondary metabolites, hypericin and hyperforin, are among the most intensively studied bioactive compounds (Kusari *et al*., 2015; Zobayed *et al*., 2006). Whereas hyperforin is contained in translucent (or pale) glands (Ciccarelli *et al*., 2001; Soelberg *et al*., 2007) which give the leaf a perforated appearance and hence the species its scientific name, hypericin on the other hand accumulates in so‐called dark glands (DGs) which differentiate in leaves (Zobayed *et al*., 2006) and other tissues (Figure S1). Hypericin is considered a promising agent for cancer photodynamic therapy (Dudek‐Perić *et al*., 2015; Garg *et al*., 2010, 2012) being classified as a type II immunogenic cell death (ICD) inducer (Krysko *et al*., 2013). By targeting the endoplasmic reticulum, hypericin induces cellular damage and apoptosis signalling (Krysko *et al*., 2013). Additionally, hypericin may also function as an inhibitor of β‐amyloid fibril formation which has opened applications in the treatment of Alzheimer's disease (Bramanti *et al*., 2010; Sgarbossa *et al*., 2008). It has been shown that crude extracts of *H. perforatum* can reduce the memory impairment in amyloid precursor protein (APP)‐transgenic mice (Hofrichter *et al*., 2013).

Despite its importance, knowledge about the biosynthesis of hypericin (Figure S2) is still incomplete (Karppinen *et al*., 2008, 2016; Kimáková *et al*., 2018; Soták *et al*., 2016a). An octaketide synthase (OKS; Karppinen and Hohtola, 2008) catalysing the condensation of acetyl‐CoA with seven malonyl‐CoAs yields octaketide products, but not the expected cyclic forms (Karppinen *et al*., 2008). For the Hyp‐1 protein (Bais *et al*., 2003), initially thought to catalyse the dimerization of emodin anthrone and emodin, as well as further C‐C bond formation between the naphthodianthrone halves, it has been shown that the mRNA is expressed in *H. perforatum* tissues unrelated to dark glands (Karppinen *et al*., 2016). Therefore, based on mRNA expression data Soták *et al*. (2016a) suggested that these functions are encoded by the POCP genes (Figure S2), but did not provide any functional data for the respective proteins. Very recently, Kimáková *et al*. (2018) raised doubts on emodin anthrone and emodin as intermediates and suggested an important role for skyrin in hypericin biosynthesis. The ultimate accumulation of hypericin within dark glands is surrounded by even more uncertainty, except some structural hints on vesicle transport by Onelli *et al*. (2002).

Though the presence of DGs correlates with hypericin content (Zobayed *et al*., 2006), the triggers towards the initiation and ultimate differentiation of these organs are unknown. Leaves are the model organ for most studies on hypericin and DGs (Onelli *et al*., 2002; Soták *et al*., 2016a,b). Highest concentrations of hypericin, however, are found in the flowers (Hevia *et al*., 2002), and pistils may be far more suited to study both hypericin biosynthesis and the formation of DGs which differentiate from the placental tissue.

By phenotyping 93 accessions, we discovered a polymorphism consisting of glanded (G+) and glandless (G−) placental tissues (PTs). The glands in the placenta can reach a very high density and, in contrast to their foliar counterpart, differentiate much later in development. The correlation between dark glands and hypericin accumulation was validated by fluorescence and FTIR microscopy. The late development of placental DGs enabled developmental studies in which pre‐ and post‐DG differentiation stages were compared. This is the first time that development of dark glands is addressed at the molecular level. Metabolomics and transcriptomics data identified novel compounds associated with DGs as well as regulatory genes associated with the development of these organs and provided novel candidate genes associated with hypericin biosynthesis. Our study demonstrates that the placenta of *H. perforatum* is a novel highly sensitive model tissue for the study of DGs and associated biosynthetic pathways and provides novel insights into these processes.

## Results

### A polymorphism for dark gland development in placental tissue

**Figure 1:**
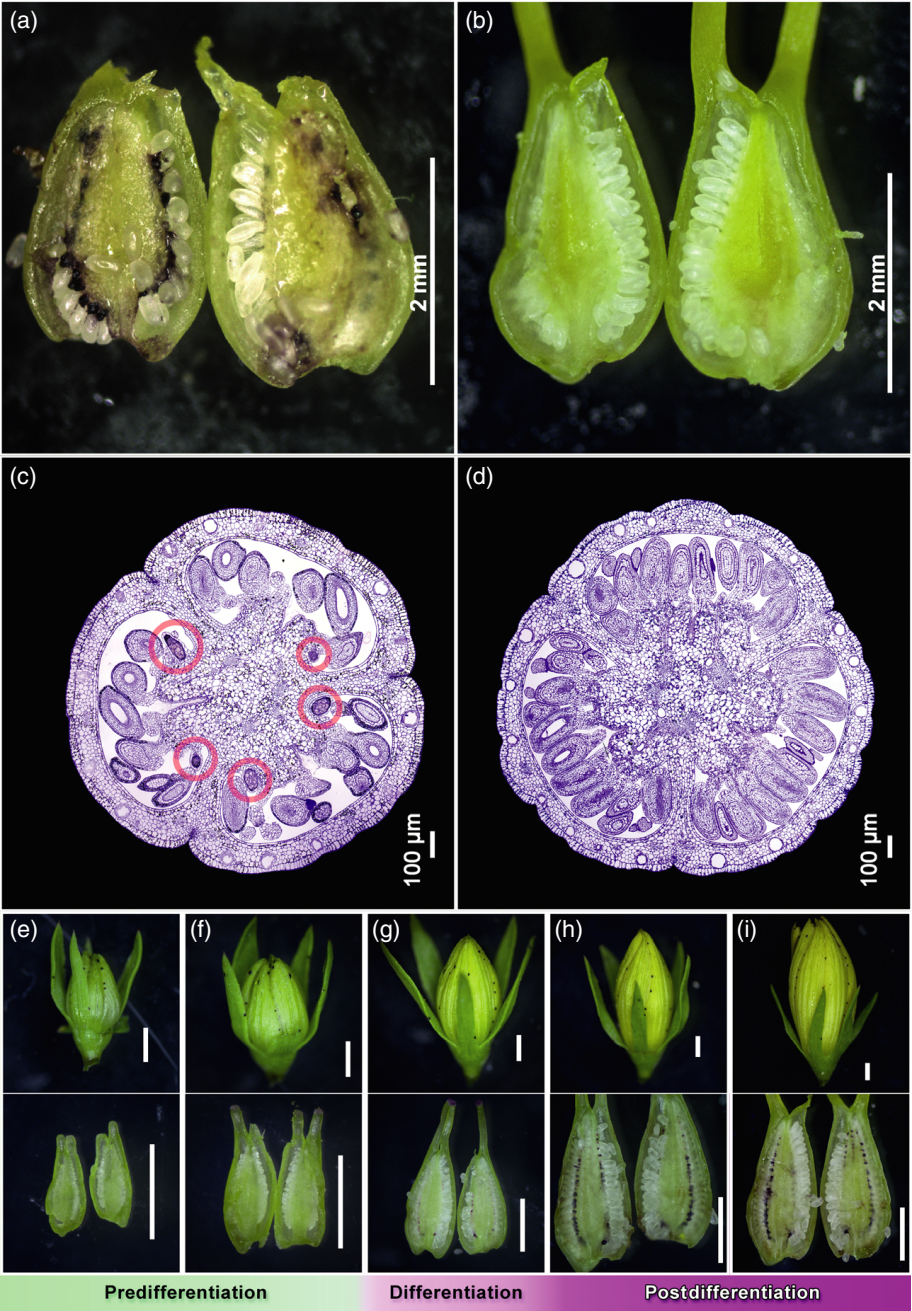
Sections of *Hypericum perforatum* pistils (a to d) and dissections of pistils from multiple stages of a G++ PT genotype (e to i). (a) Glanded placental tissue phenotype (G++ PT) from genotype HyPR‐05; (b) glandless placental tissue phenotype (G‐ PT) from genotype H06‐3251. Scale bars = 2 mm. (c,d) High‐resolution comparison of two pistil transverse sections from open flower stage. G‐ PT (right): glandless placental tissue phenotype where no dark glands are present in any part of the placental tissue (or of the entire pistil section). G++ PT (left): glanded placental tissue phenotype; here 5 dark glands (highlighted in red) are visible on the surface of the placental tissue. Scale bars = 100 μm; upper panel (e–i): flower buds; lower panel (e–i): corresponding dissected pistils; (e–f) no dark glands in pistils, flower buds 2.82 mm and 3.23 mm long, respectively; (g) differentiation of dark glands in pistils, flower bud 5.17 mm; (h–i) developed dark glands in pistils, flower buds 6.76 mm and 9.22 mm, respectively. Scale bars in (e–i) = 1 mm.

Dark glands occur in most organs of *H. perforatum* (Figure S1), except roots under natural conditions, although hormone‐induced dark glands in lateral root cultures are reported by Murthy *et al*. (2014). However, with few exceptions (Hölscher *et al*., 2009) their presence in pistils is usually overlooked. *H. perforatum* has a compound pistil composed of three carpels with parietal‐to‐axial placentation. Carpels with parietal placentation show that ovules arise submarginally whereas dark glands arise marginally (Figure 1) as they do in related organs like leaves, petals and sepals (Figure S1).

The 93 analysed accessions show a large variation in the occurrence of placental DGs (Figure S3). Twenty‐one genotypes with on average >40 of dark glands per PT were classified as carrying heavily glanded placentas (G++ PT; Figure S3), while 40 genotypes lacking DGs completely were classified as G‐ PT. The glanded and glandless phenotypes were confirmed by histological studies (Figure 1c,d) which showed that the G‐ phenotype was not due to retarded or aborted gland development (Figure S4). The remaining 32 accessions displaying an average of 1 to 40 DGs per PT were classified as G+ PT. The most heavily glanded G++ PTs packed over 130 DGs per pistil.

G‐ accessions always display dark glands in other organs like leaves, sepals, petals and anthers. This indicates that the mechanism for DG formation is present in these genotypes, but inactive in placental tissues.

### Staging of dark gland development in the pistil for transcriptome comparisons

**Figure 2:**
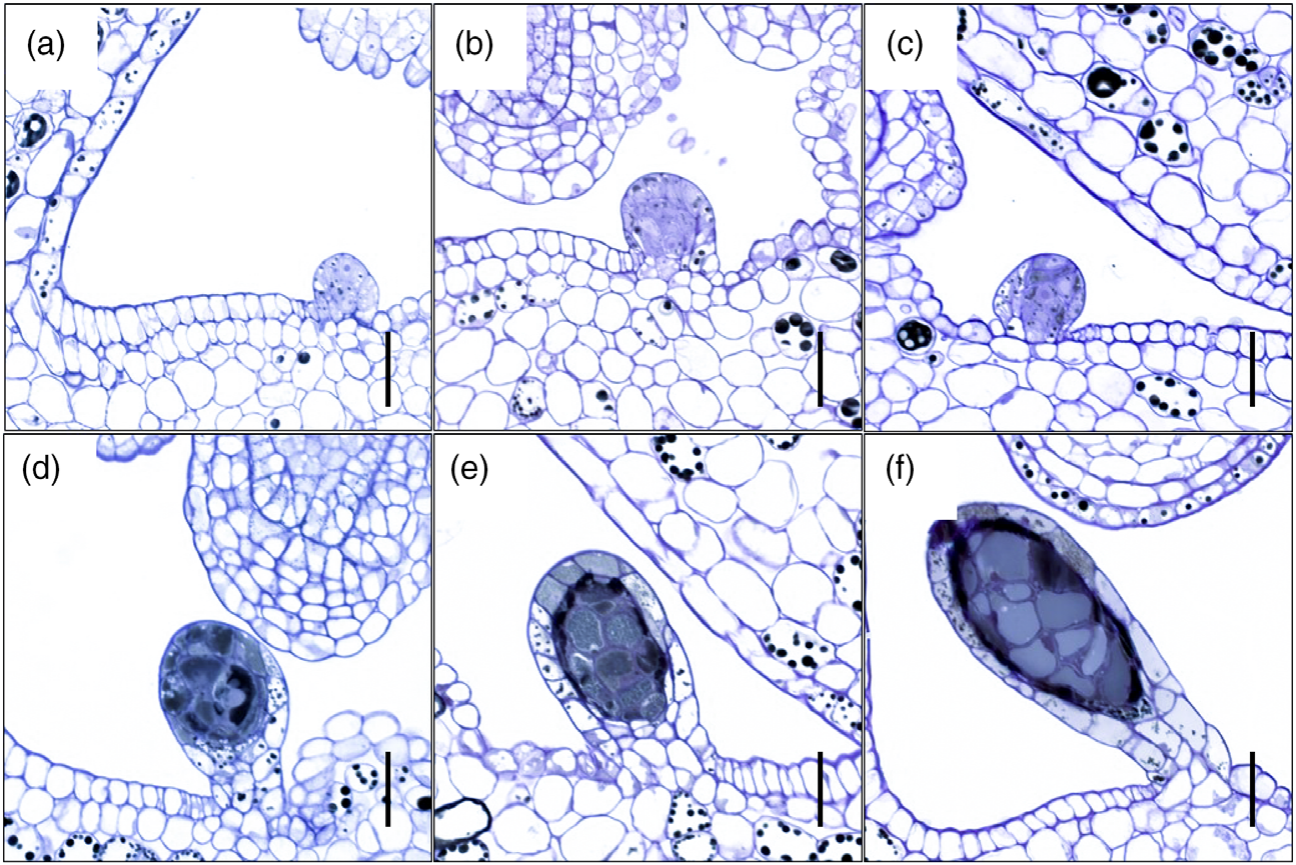
Development of dark glands differentiating from placental tissues of *Hypericum perforatum* (genotype: H06‐1460). (a) Dark gland primordium protruding from the placental tissue of a 5.0‐mm‐long flower bud; (b) dark gland differentiation from the placental tissue of a 5.5‐mm‐long flower bud; (c) dark gland differentiation from the placental tissue of a 5.7‐mm‐long flower bud; (d) growing and filling of a dark gland differentiated from the placental tissue of a 6.1‐mm‐long flower bud; (e) growing and filling of a dark gland differentiated from the placental tissue of a 8.1‐mm‐long flower bud; (f) fully differentiated dark gland protruding from the placental tissue of an open flower. Scale bars = 25 μm.

First indications of placental DGs were found when flower buds (FB) reached ~4.5 mm in length (Figure 1g). The early‐stage DGs reveal themselves as a bud‐like protrusion consisting of both epidermal and underlying parenchymal cells in the marginal area of the carpel (Figure 2a). As the glands develop and increase in size, they become darker (Figure 2b,c) and eventually assume a typical stalked morphology (Figure 2d,e). It is not until flowering that dark glands are fully developed (Figure 2f) consisting of a central area made up of large parenchymal cells delimited by a single layer of smaller parenchymal cells and a single‐layered epidermis. Compared to the foliar DGs which are invaginated in the parenchyma (Onelli *et al*., 2002), the placental DGs are fully protruded. Based on these results, three developmental phases can be defined: predifferentiation (FB shorter than 4.5 mm; Figure 1e,f), differentiation (FB 4.5–5.5 mm; Figure 1g) and postdifferentiation (FB > 5.5 mm; Figure 1h,i). This classification was used for the subsequent multistage transcriptomic study performed here.

### Hypericin accumulates during placental DG development

**Figure 3:**
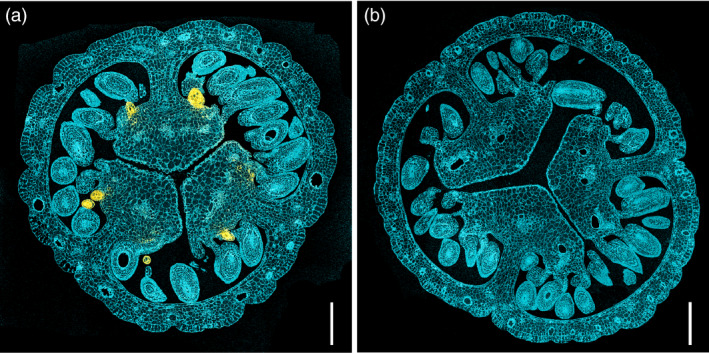
Hypericin localization through confocal microscopy; (a) pistil section of a G++ PT from genotype H06‐1998; (b) pistil section of the G‐ PT line H06‐1960 showing no hypericin fluorescence. Scale bars: 200 μm.

Spectrophotometric fingerprinting using confocal laser scanning microscopy (CLSM) proved a reliable method investigating pistils for the presence of hypericin or hypericin‐like molecules. Following a 543 nm excitation, these compounds display a peculiar fluorescence spectrum (Yamazaki *et al*., 1993). This allowed for a fast screening of fresh material and verified a positive correlation between dark glands and the presence of hypericin‐like signals. Spectrophotometric analysis of semi‐thin sections of embedded pistils showed an accumulation of hypericin‐like compounds in or near the dark glands (Figure 3a). G‐ PT sections did never show any hypericin‐like fluorescence (Figure 3b).

### Hypericin is localized inside the placental DGs

In aqueous solutions, hypericin forms aggregates with strong π–π stacking that are nonfluorescent (Falk and Meyer, 1994). Spectrophotometric analysis may only detect a lipid‐incorporated subfraction of hypericin (Ho *et al*., 2009) giving a distorted localization pattern. To circumvent this problem, hypericin distribution was therefore also studied by Fourier transform infrared (FTIR) microspectroscopy. FTIR was performed to identify chemical features of dark glands associated with the hypericin spectral fingerprint. A spectral band in the region of 1535–1485 cm^‐1^ (Figure S5f) was found to be specific for dark glands (Figure 5g,i). Principal component analysis demonstrated that this signal was caused by C=C stretching vibrations of quercetin and hypericin‐like compounds related to their polyphenolic or polyaromatic ring systems (Figure S6d). The first and fourth principal component loading spectra show that hypericin‐related features are strongly associated with dark gland chemotypes as well as a strong reduction in amide I and II bands which are associated with the abundance of proteins/enzymes. Nonglanded tissue regions (Figure S5e) did not present the distinctive fingerprint as they form separate clusters in the score plot (Figure S6a). This indicates that these polyaromatic features present in hypericin are exclusively localized in the dark glands with highest signals found in the middle of dark glands. The FTIR spectra of dark glands from PTs and from leaves were very similar and can be considered as a spectral fingerprint of these organs (Figure S6a,b).

### Metabolic validation and characterization of the dark gland's presence–absence phenotypes

**Figure 4:**
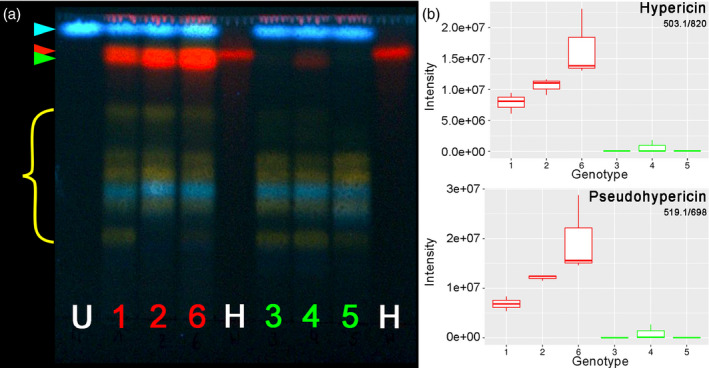
Analytical detection of hypericin and pseudohypericin in G++ PT (red: 1 = H06‐1988, 2 = HyPR‐03, 6 = HyPR‐01) and G‐ PT genotypes (green: 3 = H06‐1489, 4 = H06‐1369, 5 = H06‐3220). (a) Thin‐layer chromatogram including the internal standard umbelliferone (blue arrow) (U, 8 μg/mL) and hypericin (red arrow) (H, 1 mg/mL). Green arrow marks pseudohypericin. (b) Boxplots of MS intensities: hypericin (feature 503.1/820) and pseudohypericin (feature 519.1/698). Each boxplot is based on three biological replicates each composed of 10 pistils of the same genotype.

TLC analysis confirmed the presence of hypericin in extracts from G++ accessions and its absence from G‐ accessions (Figure 4a). In the G++ accessions, the hypericin band (Table 1, No. 8, *R*_f_ = 0.87) was accompanied by a further prominent signal with red fluorescence, which was assigned to the closely related pseudohypericin (2, *R*_f_ = 0.81). This additional signal was missing from the G‐ accessions with exception for a weak band in genotype H06‐1369 ( **2**, Figure 4a). This result is in accordance with the assumption that naphthodianthrones are restricted to dark glands and should therefore be absent in G‐ pistils.

**Table 1:** Extract from the highest positive and negative correlating features determined by PLS analysis of UHPLC‐ESI‐HRMS metabolite profiles of glanded versus glandless *Hypericum perforatum* pistils (excluding isotopes), sorted by the variance of the feature that is explained by component 1 (Var1). Due to low concentration, not all analytical parameters could be determined (−). RDB *=* ring double bond equivalent

No	Var1	Rt [s]	abs max [nm]	*m/z*[M‐H]^−^ observed	Fragment ions	Δppm	Formula [M‐H] ^−^	RDB	Assignment
1	0.941	599	–	525.1185	–	−1.077	C_30_H_21_O_9_^‐^	20.5	Hydroxyemodin dianthrone or hydroxypenicilliopsin
2	0.939	698	283 331 366	519.0711	519.0716 (100%) 487.0454 (11%) 503.0403 (5%)	−2.052	C_30_H_15_O_9_^‐^	23.5	Pseudohypericin
3	0.933	348	–	449.1081	241.0503 (20%) 285.0400 (100%) 323.0768 (7%)	−1.792	C_21_H_21_O_11_^‐^	11.5	–
4	0.931	501	–	313.0348	269.0463 (100%)	−1.808	C_16_H_9_O_7_^‐^	12.5	Endocrocin
5	0.928	776	–	505.0929	–	0.018	C_30_H_17_O_8_^‐^	22.5	Protohypericin
6	0.924	664	285 333	699.1354	519.0712 (100%)	−0.233	C_36_H_27_O_15_^‐^	23.5	Skyrin glucoside
7	0.923	669	285 333	669.1250	519.0729 (100%)	0.017	C_35_H_25_O_14_^‐^	23.5	Skyrin‐xylopyranoside/riboside
8	0.917	820	–	503.0768	503.0767 (100%) 459.0870 (8%)	−0.896	C_30_H_15_O_8_^‐^	23.5	Hypericin
9	0.914	455	–	499.0849	–	5.097	C_31_H_15_O_7_^‐^	24.5	–
10	0.910	426	–	531.0540	–	−5.403	C_27_H_15_O_12_^‐^	20.5	–
11	0.905	191	288 309	337.0923	119.0503 (5%) 163.0400 (100%) 173.0455 (5%) 191.0559 (7%)	−1.663	C_16_H_17_O_8_^‐^	8.5	O‐*p*‐coumaroylquinic acid
12	0.904	684	–	509.1240	–	−0.336	C_30_H_21_O_8_^‐^	20.5	Emodin dianthrone or Penicilliopsin
13	0.897	172	288 309	337.0921	119.0503 (5%) 163.0400 (100%) 173.0455 (5%) 191.0559 (6%)	−2.197	C_16_H_17_O_8_^‐^	8.5	O‐*p*‐coumaroylquinic acid
14	0.895	445	368	463.0877	301.0348 (100%)	−1.035	C_21_H_19_O_12_^‐^	12.5	Quercetin glycoside
15	0.892	425	266 350 368	447.0922	301.0350 (100%) 284.0402 (3%)	−2.471	C_21_H_19_O_11_^‐^	12.5	Quercetin 3‐*O*‐rhamnoside
16	0.891	461	368 271	301.0353	151.0037 (83%) 178.9985 (100%) 193.0141 (5%) 257.0454 (11%) 273.0403 (13%)	−0.119	C_15_H_9_O_7_^‐^	11.5	Quercetin
17	−0.925	727		331.19105	259.0974 (5%) 219.0665 (7%) 194.0591 (89 %) 287.2014 (100%)	−1.306	C_20_H_27_O_4_^‐‐^	7.5	Phloroglucinol derivative
18	−0.780	399	254 365	609.1455	301.0350 (100%) 271.0248 (5%) 343.0457 (7%)	−1.014	C_27_H_29_O_15_^‐^	13.5	Rutin

To gain additional information, metabolite profiles of these extracts were obtained by UHPLC‐ESI‐HRMS/PDA (Porzel *et al*., 2014). Supervised PLS‐DA was used to distinguish between the differentially glanded lines. The evaluation of the RMSEP plot (root‐mean‐squared error of prediction; Figure S7a) indicates that the first two components sufficiently describe the model. The score plot, shown in Figure S7b, confirms a good separation of the two phenotypes based on component 1. The most promising features that contribute, according to the correlation loading plot (Figure S7c), to the separation are described in Table 1, Tables S2 and S3 (numbers in bold in this section refer to the first column of Table 1). Statistical tests conducted on the selected correlated features from Table 1 are reported in Table S4. Intensity boxplots (Figure S8) display the significant difference of these features between G++ and G‐ PT lines (*P* < 0.0001; Table S2). In accordance with the TLC results, the amounts of hypericin (8) and the related naphthodianthrone pseudohypericin (2) as well as the precursor compounds penicilliopsin or emodin dianthrone (12), hydroxypenicilliopsin or hydroxyemodin dianthrone (1), protohypericin (5) and protopseudohypericin (19) are significantly higher in G++ PTs than in G‐ PTs (Figures 3b and S8). These findings are in line with the detection of hypericin and related phytochemicals in dark glands of *Hypericum* species with different MS imaging techniques (Kucharíková *et al*., 2016; Kusari *et al*., 2015). The elevated feature with m/z 313 and retention time 501 s was identified as endocrocin (4). This anthraquinone differs from emodin, the proposed monomeric precursor of hypericin, by a carboxyl group. Moreover, two glycosides of the dimeric anthraquinone skyrin (6 and 7) are equally increased in the G++ PT materials. Surprisingly, the flavonol quercetin (16), some quercetin glycosides (14 and 15) and phenolic acids such as O‐*p*‐coumaroylquinic acid (11 and 13) are also more abundant in glanded tissues.

To identify further differentiating features, G‐ PT samples were checked for analogous concentration increases (Table S3). Among the compounds identified this way was a phloroglucinol derivatives (17) and the quercetin diglycosid rutin (18) (Table 1, negatively correlating features). The other contributing features are not identified, but their chromatographic behaviour suggests highly apolar compounds possibly related to phloroglucinols or terpenoids.

### Transcriptome‐based genetic subtraction of glandless vs glanded placental tissues identifies both known and novel genes associated with hypericin biosynthesis and dark gland development

To reveal genes involved in DG development and the synthesis of hypericin as well as other metabolites correlated with DGs, mRNAs were isolated from placentas of three G‐ (H06‐1369, H06‐1489 and H06‐3251) and three G++ lines (HyPR‐05, HyPR‐09 and H06‐1988) at predifferentiation (FB25), differentiation (FB45) and postdifferentiation stages (FB75; experimental design described in Figure S10). After preparation of strand‐specific cDNA libraries (Lexogen SENSE), all samples were sequenced on a HiSeq 2500 (Illumina). Between 7.8 and 24.8 mio reads (average 16.4 ± 4.1 mio) were obtained per library and combined after adapter and quality trimming for a Trinity‐based assembly (Grabherr *et al*., 2011), which yielded 82,122 gene fragments with 204,735 isoforms. Trimmed reads of each library were mapped onto this assembly using Kallisto (Bray *et al*., 2016), and read counts for all isoforms of a gene fragment were added to obtain an expression value for the respective gene fragment. Using the R package DESeq2 (Love *et al*., 2014), pairwise comparisons between different stages (FB25, FB45, FB75) and line types (G−, G++) were made, using all lines of a given type and stage as replicates (Figure S9a,b; Table S5). A first approach was taken by comparing developmental stages within G− and G++ lines (Figure S9a, red and green). This approach identified 115, 1757 and 3510 differentially expressed genes (DEGs) in G‐ lines for the comparisons FB45/FB25, FB75/FB45 and FB75/FB25, respectively, resulting in 3665 DEGs when united. For G++ lines, the same comparisons identified 160, 273 and 2010 DEGs, resulting in 2018 DEGs when united. Comparing the set of 3665 DEGs from the G‐ comparisons with the 2018 DEGs from the G++ comparisons (Figure S9b), 462 DEGs were identified, which occur in G++ comparisons, but not in G− comparisons, and therefore are potentially related to DG development (Figure S9b, yellow). An even more restrictive set of 67 DEGs specific for DG development was obtained by identifying the overlap between the 462 DEGs and a set of 811 DEGs identified by comparing G++ and G− line stages directly (Figure S9b, blue; FB25: 311 DEGs which were not considered, since no DGs exist at this predifferentiation stage; FB45: 351 DEGs; FB75: 725 DEGs; united: 841 DEGs). Figure 6 provides the expression profiles of these 67 DEGs after k‐means clustering (6 clusters) and shows that DEGs in clusters 1–3 are strongly up‐regulated during DG development, 24 DEGs in cluster 4 are slightly up‐regulated, and expression levels of 10 DEGs in clusters 5 and 6 are slightly lower (down‐regulated) in G++ lines than in G‐ lines. A preliminary annotation of the 67 DEGs included in these six clusters is provided in Table S6, and log2 fold change values, adjusted *P*‐values, and read counts and normalized read counts are given in Table S5.

The fact that 10 DEGs in cluster 1 show strong up‐regulation already at the differentiation phase (FB45) suggests that regulatory genes might be found in this group. Inspection of these DEG sequences reveals an AGL6 homologue and a MYB transcription factor (homologue to MYB38 from *Arabidopsis thaliana*).

DEGs up‐regulated later during DG development (postdifferentiation, stage FB75) as in clusters 2 (13 DEGs) and 3 (10 DEGs) are expected to contain genes for the synthesis of hypericin and other metabolites or accessory functions. Three DEGs in cluster 2 align almost perfectly (except eventually unspliced intron sequences) with different parts of HpPKS2 (EF186676, HQ529467, EU635882), the octaketide synthase (OKS) responsible for the first dedicated step in hypericin biosynthesis (Karppinen *et al*., 2008). The PR‐10‐related genes (POCP 1‐3; KU744669, KU744670, KU744671) previously discovered to be differentially expressed in DG‐containing tissues (Soták *et al*., 2016a) are represented by 4 DEGs in cluster 2 (Table S6).

Cluster 3 contains 3 DEGs, which encode at least two ABC‐transporter genes of type G (see https://www.ebi.ac.uk/interpro/entry/IPR034003), which might be involved in metabolite transport.

## Discussion

### Placental tissue as a novel model organ to be used for the study of dark gland development and related biosynthetic processes

*Hypericum perforatum* has been used as a medicinal plant since ancient times. One of its major secondary metabolites, the phototoxic hypericin, is confined to conspicuous so‐called dark glands which are present in many organs (Kucharíková *et al*., 2016; Kusari *et al*., 2015; Zobayed *et al*., 2006), and its potential as an anticancer agent is of economic and scientific interest (Agostinis *et al*., 2000, 2002; Garg *et al*., 2010, 2012; Krysko *et al*., 2013; Morgan and Oseroff, 2001). Though the flowers are the most potent part of *H. perforatum* (Hevia *et al*., 2002), research on hypericin biosynthesis nearly exclusively focused on the leaf (Fornasiero *et al*., 2013; Onelli *et al*., 2002; Soták *et al*., 2016a,b). Since leaves without dark glands are unknown in *H. perforatum*, it has been common practice to separate leaf lamina from leaf rim to compare between glanded and glandless tissues (Soták *et al*., 2016a,b). The results of the present study suggest, however, that placental tissue is a superior model organ for the investigation of hypericin biosynthesis and dark gland development. We have identified accessions with an average of >120 DGs per PT next to accessions never developing DGs in the same tissue enabling ideal comparative analyses. A peculiarity of placental DGs is their late development, which does not start before flower buds are ~4.5 mm long (FB45). This makes predifferentiation stages more accessible than in leaves where DGs differentiate very early (Curtis and Lersten, 1990; Fornasiero *et al*., 2013).

The placental DGs differentiate along the margins of carpels, a position corresponding to the leaf margin as do most other locations in which DGs occur in *H. perforatum*, such as sepal and petal rims as well as anther tips (Figure S1) since all these organs are modified leaves (Coen and Meyerowitz, 1991; Honma and Goto, 2001; Pelaz *et al*., 2001; Yan *et al*., 2016). Following this logic, the placental DGs can be considered as a remnant of leaf rim in the carpels that have been lost in G‐ accessions, although deeper phylogenetic studies are needed to test such a hypothesis.

Confocal microscopy and FTIR microscopy confirm the presence of hypericin(‐like) signals in dark glands and the absence of these signals in glandless PTs.

**Figure 5:**
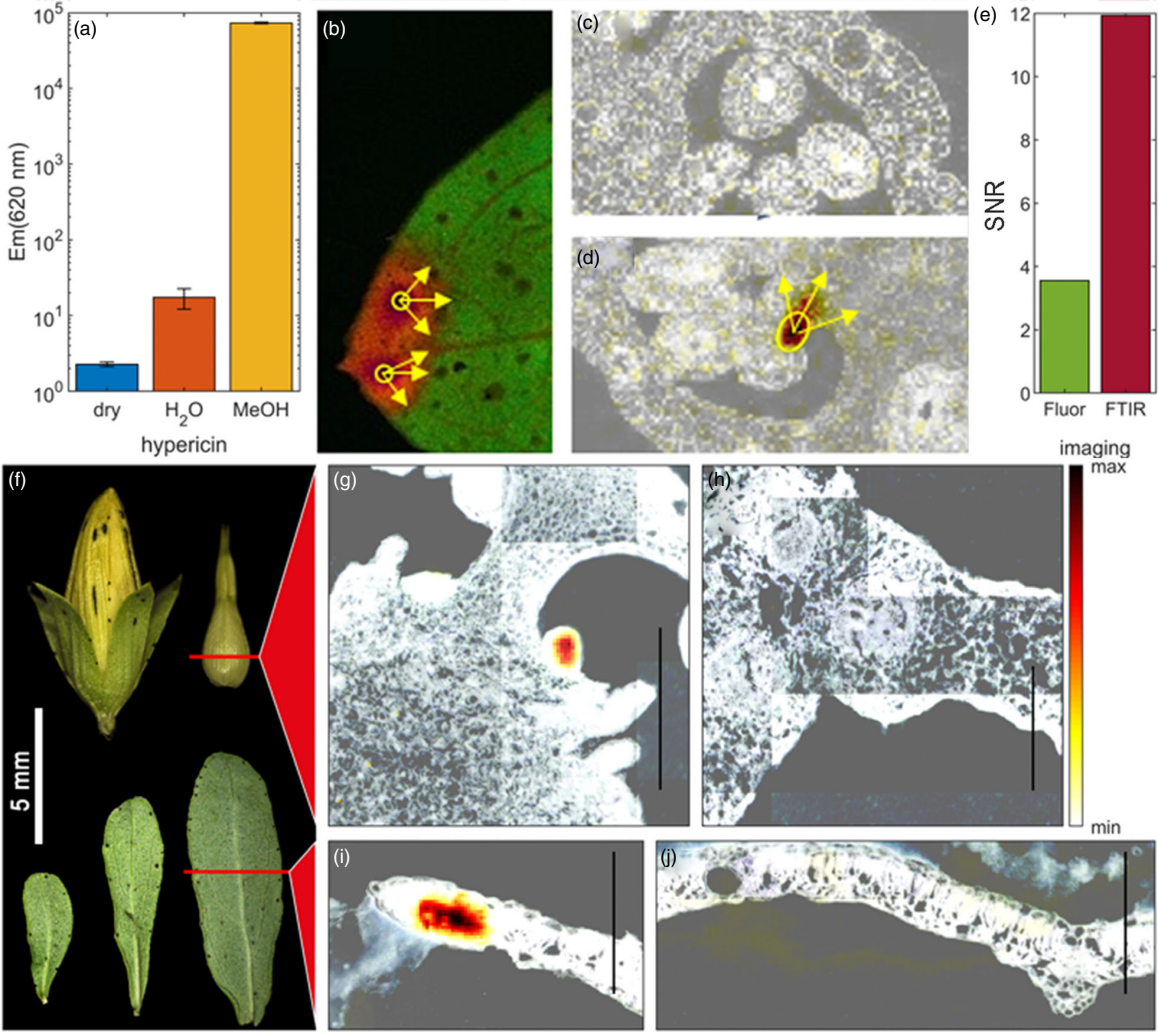
Aromatic specific signal strictly associated with dark glands in placental tissues and leaves of *Hypericum perforatum* through confocal microscopy imaging (from a to d) and FTIR microscopy (from g to j). (a) Fluorescence emission of pure hypericin in its dry state and in water or methanol environment. (b, d) Confocal microscopy pictures of fixed leaf and section of G++ PT pistils of *H. perforatum* showing fluorescence signal from dark glands spread into adjacent tissues due to the fixation process; (c) unrelated fluorescence in G‐ PT pistil. (e) Signal‐to‐noise ratio of FTIR‐ and fluorescence‐based imaging for dark gland chemotype; (f) flower and leaf of *H. perforatum* showing regions of tissue sections; (g) FTIR imaging of placental dark glands in a G++ PT genotype; (h) FTIR imaging of a placental tissue in a G‐ PT genotype; (i) FTIR imaging of a leaf‐rim section containing a dark gland; (j) FTIR image of dark glandless central leaf region showing no specific signal. FTIR images are presented as an overlay of a corresponding structural image (black and white) and the colour‐scaled integration intensity image of polyaromatic C=C stretching vibrations. Fluorescence pistil images show structure in black and white overlaid with fluorescence intensity using the same colour map as in FTIR images. Yellow arrows show signal spreading due to sample treatment into adjacent tissues of the dark glands. Scale bar = 250 μm.

While conventional fluorescence imaging only allows testing for presence of hypericin, FTIR imaging can detect and localize the chemical fingerprint associated with dark glands in a semiquantitative manner by simple spectral band integration along with a higher signal‐to‐noise ratio (Figure 5e). Fluorescence imaging is impaired by quenching through π–π stacking in agglomerations of hypericin, which can be reduced through tissue fixation, but yields preparation artefacts (Figure 5c,d). FTIR can be applied to dried cryosections, which do not change the chemical composition or location of relevant metabolites within the tissue, since only water is removed by lyophilization prior to imaging. These encouraging results provide a new semiquantitative tool to investigate dark gland development related to its chemical constituents as well as a first step towards a comparative chemical analysis with other tissues in *Hypericum*, for example translucent glands at a topological scale.

Comparative analysis of metabolites in G‐ and G++ accessions using UHPLC‐HRMS shows that hypericin, pseudohypericin, protohypericin and quercetin and its rhamnoside, all of which have been found within DGs (Kucharíková *et al*., 2016; Kusari *et al*., 2015; Zobayed *et al*., 2006), correlate with the presence of DGs in the placental tissue of *H. perforatum*. Also, skyrin glycosides, observed by Wirz *et al*. (2000) and more recently by Kimáková *et al*. (2018) in *Hypericum* extracts, are correlated with the presence of DGs. If interpreted as side products of hypericin synthesis, these compounds suggest that biosynthesis of hypericin proceeds preferably via initial formation of the C5‐C5’ bond between the naphthodianthrone halves (Figure 7) and not via the C10‐C10’ bond as postulated by Soták *et al*. (2016a). Already Falk (1999) has considered the C5‐C5’ bond formation as an alternative to an initial C10‐C10’ bond. Since emodin dianthrone and its hydroxylated derivative (Table 1, No. 12 and No. 1), which indicate a C10‐C10’ bond, possess the same molecular formulas as penicilliopsin and hydroxypenicilliopsin with a C5‐C5’ bond, respectively, we cannot differentiate between these substances in the absence of reference compounds defining the retention time on the UHPLC C18 column. Currently, we are also not able to define whether emodin anthrone, emodin and atrochrysone (as proposed by Gill and Giménez (1991) for the synthesis of austrovenetin) are the substrates for this reaction, since none of these potential intermediates could so far be detected in our analyses.

### Identification of genes involved in dark gland development and associated biosynthesis processes

**Figure 6:**
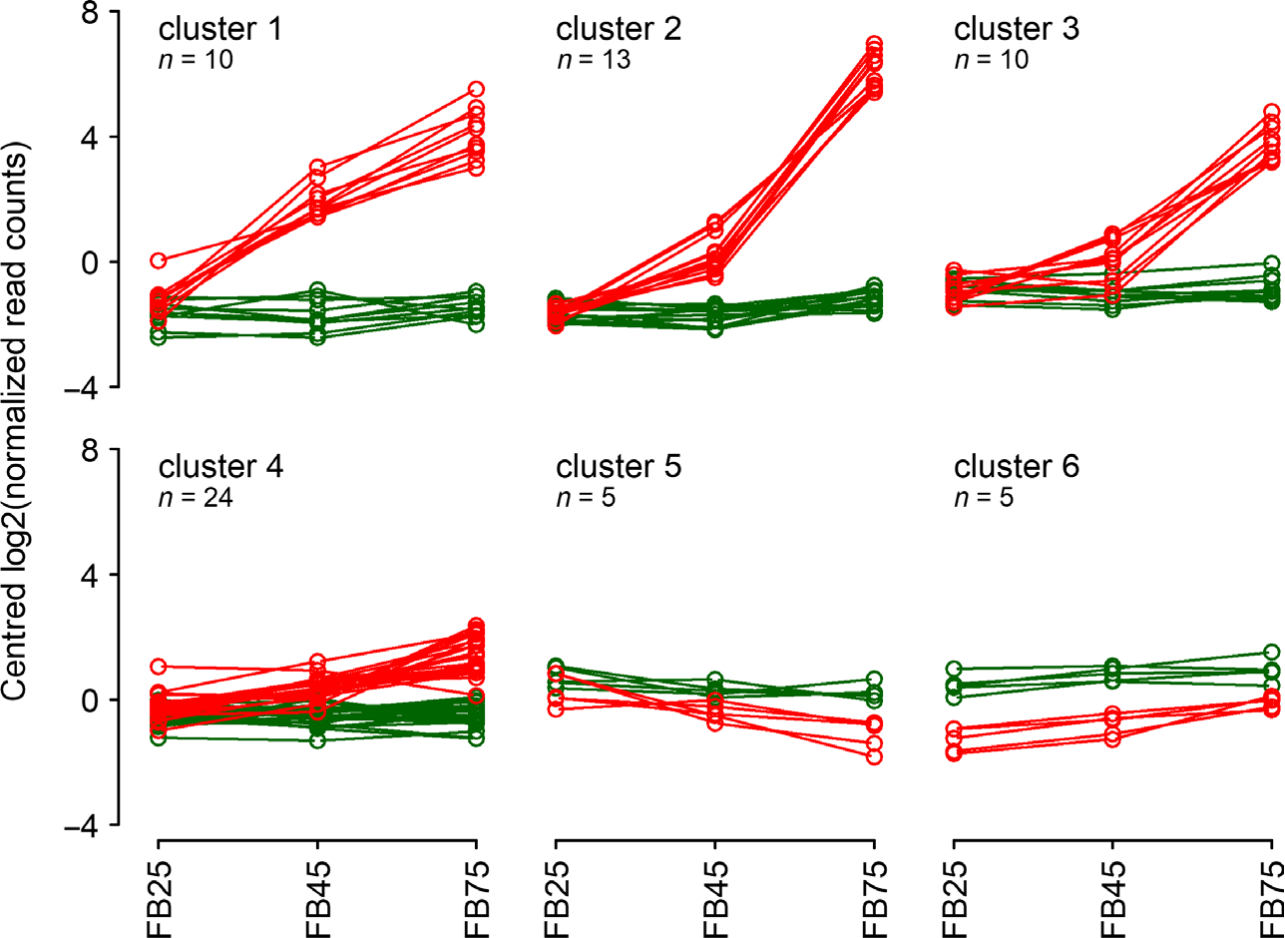
Sixty‐seven DEGs associated with dark gland development and biosynthetic processes, distributed in 6 k‐means clusters. X‐axes represent developmental stages: predifferentiation (FB25), differentiation of DGs (FB45) and postdifferentiation (FB75). Green = expression in glandless placental tissues (G‐ PT); red = expression in glanded placental tissues (G++ PT).

The main aim of this study was to identify putative regulators of dark gland differentiation and candidate genes involved in secondary metabolism, which are expected to show a strong up‐regulation already at the developmental stage FB45 of G++ placental tissues. Here, we discuss 33 DEGs in clusters 1 to 3 only (Figure 6 and Table S6), since these are highly induced during differentiation (FB45) and maturation stages (FB75) of dark glands, while 34 DEGs in clusters 4 to 6 are only weakly induced (24 DEGs, cluster 4) or slightly repressed (5 DEGs, cluster 5) during DG development or at all analysed stages (5 DEGs, cluster 6).

With respect to biosynthesis of flavonoids, it is noteworthy that a 2‐oxoglutarate and Fe(II)‐dependent oxygenase (2‐ODD, cluster 1; Farrow and Facchini, 2014) and a cytochrome P450 enzyme (CYP, cluster 3; Schuler and Werck‐Reichhart, 2003), which are typical for hydroxylation and dehydration reactions in that pathway, are encoded by DEGs, as well as an UDP‐glucosyl transferase (UGT, cluster 1) for the transfer of sugar moieties to flavonols (Saito *et al*., 2013), leading to metabolites such as the observed quercetin glycosides. For translocation of the cytosolically synthesized flavonoids across membranes and transport to the vacuole, mechanisms including glutathione S‐transferases (GST, clusters 1, 2 and 3; Wagner *et al*., 2002; Dixon *et al*., 2010; Labrou *et al*., 2015) and ABC transporters (ABC clusters 1 and 2; Verrier *et al*., 2008) are discussed (Zhao, 2015). Genes encoding both types of proteins are within the list of highly induced DEGs in clusters 1 to 3. In addition, a ß‐glucosidase targeted for secretion (BGLU cluster 2; Xu *et al*., 2004) and a membrane integral protein of the major facilitator superfamily (MFP cluster 3; Reddy *et al*., 2012) are observed, which suggest cleavage of sugar moieties from vacuolar or secreted compounds or cell wall components and sugar import (MFP; Figure S11). Since no further highly induced DEGs encoding transport proteins are observed, naphthodianthrone transport might be similar to flavonoid transport and may involve GST‐associated vesicles. This would confirm the observations by Onelli *et al*. (2002) who extensively reported the accumulation of vesicles in and around the developing dark glands of leaves.

**Figure 7:**
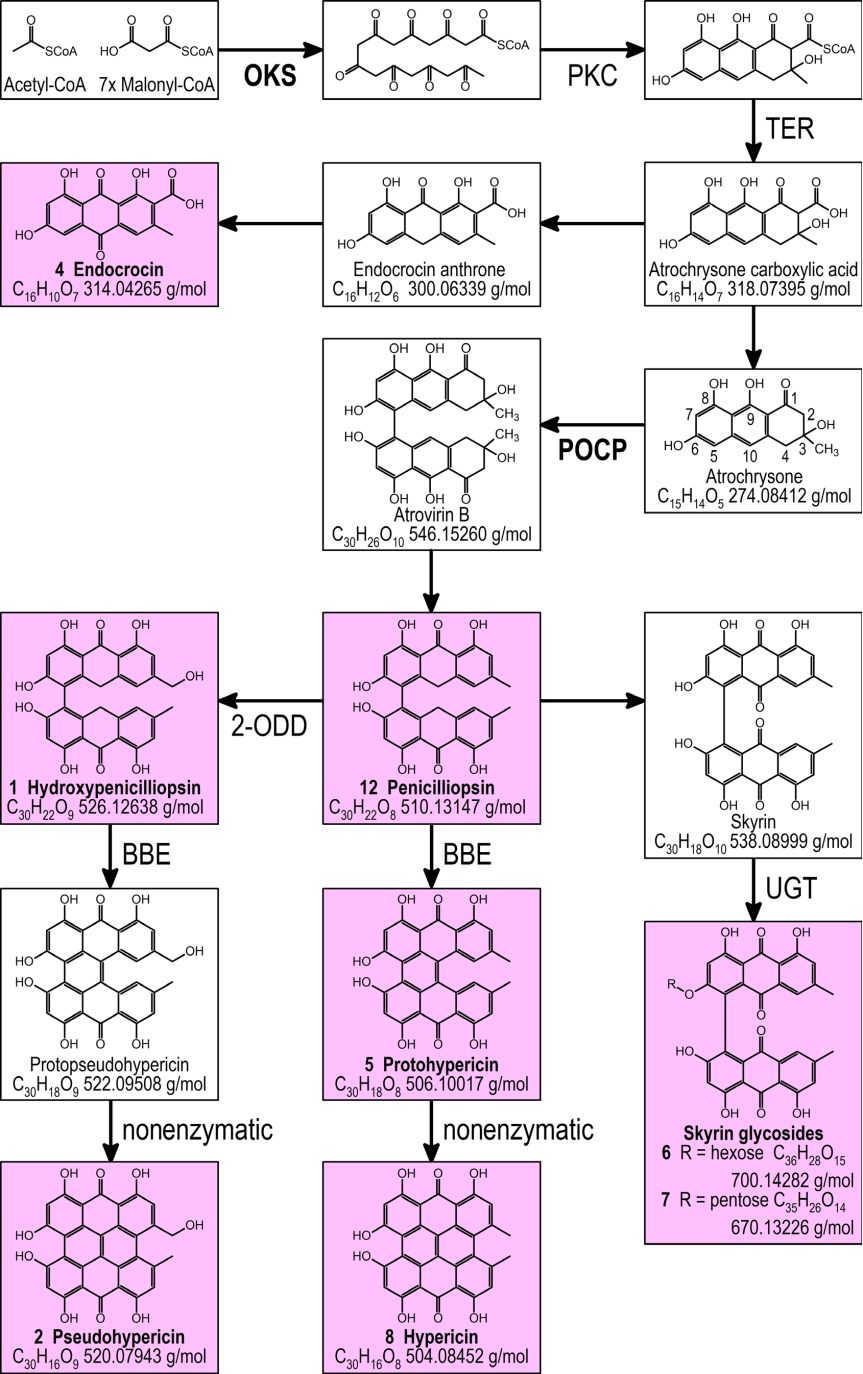
Proposed hypericin biosynthetic pathway based on our metabolomics, transcriptomics and functional annotation (given in Table S6). Compounds positively correlated with the presence of dark glands are highlighted by a pink background and accompanied by numbers in bold as given in Table 1. Enzymes shown in bold were suggested by previous literature. The numbering of the carbon skeleton according to Gill and Giménez (1991) and used throughout the text is given in the structural formula of atrochrysone. Pictured masses represent calculated exact masses.

With respect to naphthodianthrone synthesis, we observed several DEGs encoding the well‐described octaketide synthase HpPKS2 (OKS; cluster 2), executing the first committed step of hypericin synthesis (Karppinen *et al*., 2008). Remarkable in cluster 2 is the highly induced potential polyketide cyclase (PKC), which might encode a missing function for correct cyclization of the octaketide proposed by Karppinen *et al*. (2008). Two DEGs annotated as dihydrofolate reductases (clusters 2 and 3) belonging to the superfamily of serine hydrolases may encode thioesterases (TER) involved in releasing octaketide and tetraketide precursors of hypericin and flavonoids from their coenzyme A conjugates (Xu *et al*., 2004). In addition, we detected DEGs encoding phenol oxidative coupling proteins (POCPs) discovered by Soták *et al*. (2016a) to be induced in dark gland‐containing tissues and suggested to be involved in C‐C bond formation between the naphthodianthrone halves of hypericin and its derivatives. To our knowledge, no experimental proof of the functionality of the PR‐10‐related POCPs exists, but a PR‐10‐related protein has been shown to exhibit norcoclaurine synthase activity in opium poppy by performing a condensation reaction between dopamine and 4‐hydroxyphenylacetaldehyde in benzylisoquinoline alkaloid synthesis (Lee and Facchini, 2010). Besides the POCP genes, we identified a berberine bridge enzyme (BBE; cluster 2, Daniel *et al*., 2017), which suggests an additional or alternative function to POCPs for the formation of one of the three C‐C linkages between the naphthodianthrone halves of hypericin. Since the encoded BBE is targeted for secretion by a N‐terminal signal peptide (Käll *et al*., 2007), it will most likely close the last C‐C bond for which an enzyme is required, similar to THCS and CBDS in tetraketide‐based cannabinoid synthesis (Sirikantaramas *et al*., 2004; Taura *et al*., 2007), which belong to the same protein family and are predicted to contain N‐terminal signal peptides as well. Our observation that skyrin glycosides are correlated with the presence of dark glands suggests that the C5‐C5’ bond is formed first. This view is supported by the previous detection of skyrin glycosides in *H. perforatum* (Wirz *et al*., 2000) and the recent notion that their presence is correlated with occurrence of hypericin in *Hypericum* species (Kimáková *et al*., 2018). As mentioned above, initial C5‐C5’ bond formation has been suggested earlier as an alternative to C10‐C10’ bond formation (Falk, 1999). Therefore, POCPs and the berberine bridge enzyme might be involved in formation of the C5‐C5’ bond and the C10‐C10’ double bond, respectively, while the C4‐C4’ bond might be formed in a last step nonenzymatically. Since Kusari *et al*. (2015) and Kimáková *et al*. (2018) noted that the presence of emodin anthrone and emodin does not correlate with the occurrence of hypericin in *Hypericum* species, the biosynthesis of hypericin might proceed via atrochrysone, atrovirin B, austrovenetin and penicilliopsin (Figure 7; compare Gill and Giménez, 1991) completely circumventing emodin and emodin anthrone, but retaining the potential to yield skyrin.

Enigmatic are currently functions of DEGs highly induced in G++ placental tissues encoding osmotin (a PR‐5 protein; cluster 3), a membrane‐localized cytochrome b561 (electron transport through a membrane; cluster 3), and a S‐adenosyl‐L‐methionine‐dependent methyltransferase, since neither synthesis of the dark gland‐correlated flavonoids nor synthesis of hypericin and its derivatives would require the transfer of a methyl group.

When looking for genes putatively involved in DG development, we expected a differential expression to be detected already at FB45. Cluster 1 contains all the DEGs characterized by this expression pattern and includes only two transcription factors in which the up‐regulation is strictly synchronized with the beginning of dark gland differentiation.

Agamous‐like 6 (AGL6) is a MADS transcription factor known in *Arabidopsis thaliana* as a master regulator of floral organ identity and meristem fate (Ohmori *et al*., 2009). A dramatic increase in transcription of this gene is detected already at FB45 in the G++ PT genotypes, indicating a synchronization with the formation of DG primordia on the surface of the placenta. At FB75, AGL6 shows average levels of transcription in G++ lines that are at least 100 times higher than in their glandless counterpart. This implies a possible involvement of AGL6 with the differentiation of DGs in the placenta. This gene is known as one of the MADS box genes that went through a higher number of duplications and neofunctionalization in the evolutionary history of dicots (Viaene *et al*., 2010). It is possible that along the evolutionary history of *H. perforatum*, a copy of AGL6 was neofunctionalized to regulate processes involved in DG formation.

The other putative regulator included in cluster 1 is an R2R3‐Myb transcription factor. This class of transcription factors mainly regulates plant‐specific processes connected with secondary metabolism or cell fate and organ identity determination (Stracke *et al*., 2001). The best BLASTX hit of this DEG with *A. thaliana* is MYB38, also known as RAX2, a regulator of development responsible for the patterning of lateral meristem initiation (Muller *et al*., 2006). We hypothesize that this transcription factor regulates the formation of dark glands since they emerge from a specific lateral patterning of the placenta exactly like in the case of lateral meristems patterning regulated by RAX2. MYB38 belongs to the subgroup 14 of the big R2R3‐MYB family in *A. thaliana* (Stracke *et al*., 2001). Despite the fact that the R2R3‐MYB family mainly regulates either secondary metabolism or cell fate and organ differentiation (Stracke *et al*., 2001), all the members of the subgroup 14 (MYB36, MYB37, MYB38, MYB68, MYB84 and MYB87) are exclusively regulating cell fate and organ formation (Feng *et al*., 2004; Fujiwara *et al*., 2014; Liberman *et al*., 2015; Muller *et al*., 2006).

A conclusive demonstration of function for the candidates reported here must await experiments with altered gene expression profiles. Nevertheless, the known functions of these candidate genes in other species and their observed expression patterns in *Hypericum* provide the first step towards the understanding of dark gland development.

It remains unclear whether the differentiation of DGs can promote downstream biosynthetic processes or whether these processes induce the formation of DGs. Our data show that the up‐regulation of AGL6 and MYB38 starts earlier than the activation of most biosynthesis‐associated genes in clusters 2 and 3. This suggests that DG formation precedes the synthesis of hypericin and other associated compounds.

The candidates reported in this study, when combined with an efficient transformation pipeline, could be used to completely shut down or, alternatively, hyperactivate dark glands.

The production of new genotypes containing high levels of hypericin or of hypericin‐free cultivars would be highly valuable for research on depression, cancer and Alzheimer's disease where these types of *Hypericum* extracts are already in high demand.

## Experimental procedures

### Plant material and growth conditions

The 93 wild accessions of *H. perforatum* L. used in this study cover the complete range of ploidy, mode of reproduction and genetic backgrounds as determined by Koch *et al*. (2013) and Molins *et al*. (2014) (Table S1). Fifty seeds per genotype were sown 1 cm deep in 12 × 10 cm pots and kept in long‐day conditions: 16 h light, 250 μmol/s/m^2^ at 21°C; and 8 h darkness at 18°C. High humidity levels were kept by using germination capsules with translucent caps. Four weeks after sowing, seedlings were transferred into 16 × 15 cm pots and moved to a greenhouse with 16 h of light, at 300–400 μmol/s/m^2^, at 21°C, and 8 h of darkness at 17°C, at 70% average humidity. Three months after germination, plants destined for pistil phenotyping were transferred to the field. Plants used for RNAseq experiments were grown in a phytotron with 16 h of light at 400–450 μmol/s/m^2^ at 23°C, and 8 h of darkness at 18°C at 70% humidity.

### Phenotyping and histological analysis

All field‐grown accessions were analysed under a Stemi 2000 Zeiss stereomicroscope for the presence or absence of dark glands within the pistil. Three open flowers per plant and five plants per accession for a total of 1395 flowers were examined. Genotypes with up to 40 glands per PT were classified as G+ PT, and those with >40 dark glands per PT were classified as G++ PT. Genotypes without placental dark glands were classified as glandless (G‐ PT). For histological studies, isolated pistils were fixed and embedded in Spurr resin according to Rutten *et al*. (2003). Semi‐thin sections (2 μm) were cut on a Reichert‐Jung Ultracut S (Leica, Vienna, Austria), stained with crystal violet and examined in a Zeiss Axio Imager light microscope (Carl Zeiss, Jena, Germany).

### Spectrophotometric detection of hypericin

Fast spectrophotometric detection of hypericin in fresh pistil material was performed with a LSM780 confocal laser scanning microscope (Carl Zeiss). Using a 488 nm laser line for excitation, emission was recorded over the range 491–695 nm. Hypericin‐style emission profiles (Yamazaki *et al*., 1993) were unmixed from unrelated background fluorescence using the ZEN software (www.zeiss.com). For more precise localization studies, semi‐thin (2 μm) sections of Spurr‐embedded pistils that had been fixed without osmium were employed.

### FTIR imaging

Cryosections (16 μm) of pistils and leaves were prepared and imaged according to Munz *et al*. (2017) using the Bruker Tensor 27 coupled to a Hyperion 3000 microscope (Bruker Optics, Ettlingen, Germany). Absorbance images were created in OPUS (www.bruker.com) by integrating the spectral information between 1535 and 1480 cm^‐1^ and subtracting a linear baseline defined by the absorbance at 1535 and 1155 cm^‐1^. A reference baseline was introduced by the spectral minima common in plant tissue spectra at 1155 cm^‐1^ and the minima for gland tissue at 1535 cm^‐1^ allowing the exclusion of unspecific signals in the integration.

### Metabolite analyses

#### Extraction of plant material

For a comparative chemical analysis, pistils from open flowers of three glanded (G++ PT: H06‐1988, HyPR‐3, HyPR‐1) and three glandless PT genotypes (G‐PT: H06‐1498, H06‐1369, H06‐3220) were selected. For each genotype, three biological replicates comprising ten pistils each were used. After lyophilization, samples were ground in a ball mill (MM 400; Retsch, Haan, Germany) for 30 s at 30 Hz. The resulting powder was mixed with LC‐MS‐grade methanol (10 mg/mL) containing 8 μg/mL umbelliferone (HPLC‐grade; Sigma, Darmstadt, Germany) as an internal standard. After brief mixing on a vortex, extraction was continued in an ultrasonic bath for 15 min. Following centrifugation (15 min, 14,000 min^‐1^) the supernatant was used for UHPLC‐MS/PDA and TLC analysis.

#### TLC analysis

Pistil extracts (see above, replicates combined) were applied to SilicaGel plates (60 G F254, 9 × 7 cm; Merck, Darmstadt, Germany). The mobile phase comprised ethyl acetate, formic acid (Roth), acetic acid (Roth), distilled water and dichloromethane (100:10:10:11:25 V/V/V/V/V) (Ernst, 2003). After migration, plates were air‐dried and sprayed with natural product reagent (0.5 % methanolic 2‐aminodiphenylborinate; Fluka). Signals were visualized with UV light at 366 nm.

#### High‐resolution UHPLC‐MS/PDA analysis

Negative ion high‐resolution ESI mass spectra were obtained from an Orbitrap Elite mass spectrometer (Thermo Fisher Scientific, Bremen, Germany) equipped with a HESI electrospray ion source (spray voltage: 4.0 kV; source heater temperature: 325°C; capillary temperature: 300°C; FTMS resolution: 15,000). Nitrogen was used as the sheath and auxiliary gas. The MS system was coupled with an ultra‐high‐performance liquid chromatography (UHPLC) system (Dionex UltiMate 3000; Thermo Scientific), fitted with a RP‐C18 column (1.9 μm; 50 x 2.1 mm; Hypersil GOLD; Thermo Scientific; column temperature: 40°C) and a photodiode array detector (PDA; Thermo Scientific; 220–650 nm). For UHPLC separation, a H_2_O:MeOH solvent gradient system (each containing 0.1% formic acid) at a flow rate of 400 μL/min was applied (95:5 for 1 min, 10 min gradient to a ratio of 0:100, hold for 4 min, returning to 95:5 in 1 min, isocratic hold for 4 min). The data were evaluated by the Xcalibur software 2.2 (Thermo Fisher).

#### MS raw data processing and multivariate data analysis

MS data processing was performed in R with the XCMS package (version 1.52.0, bioconductor.org). Xcalibur raw output files (*.raw) were converted into standard format mzData files (*.mzML) utilizing proteowizard (proteowizard.sourceforge.net). Peak picking was performed in XCMS with centWave parameters: ppm = 10, peakwidth = c(5,12), snthr = 5 and prefilter = c(3,1500). After peak grouping (minfrac = 1, bw = 5, mzwid = 0.002), retention time correction was performed using LOESS correction and peak grouping was repeated. Missing values were filled with fillPeaks function. The final data matrix contained features (mz*RT^−1^) in rows and samples in columns. For data evaluation, an output table was used for partial least squares discriminant analysis (PLS‐DA) performed with the R package pls (version 2.6‐0). Gland presence was coded as the Y variable (G++ PT = 1, G‐ PT = 0). Results were further statistically analysed to determine significant differences in feature intensity. Homogeneity of variance was checked with an F‐test to decide the usage of the t‐test or the variance independent Welch two‐sample t‐test. Effect size and power of analysis were calculated feature‐wise. Identification of known compounds was based on exact mass of detected ions and fragmentation pattern compared to massbank.eu.

### RNA extraction

Total RNA was extracted from placental tissues at different stages of flower development as measured by flower bud (FB) length. The FB classes selected were 2.5 to 3.5 mm (FB25; dark gland predifferentiation), 4.5 to 5.5 mm (FB45; dark gland differentiation) and 7.5 to 8.5 mm (FB75; dark gland postdifferentiation). Placental tissues were isolated from the pistils and ovules removed. After initial storage on ice in a 70% ethanol solution containing 0.1% Tween‐20, samples were frozen in liquid nitrogen and stored at −80°C. For each developmental stage, PTs from at least 20 individuals were collected. The large number of PTs enabled analyses to be performed without RNA amplification steps. Samples were ground using six 3‐mm metal beads in a Retsch grinder running at 30 Hz for three minutes. RNA was extracted by InviTrap Spin Plant RNA Mini Kit following the manufacturer's instructions. Finally, total RNA was screened by an Agilent 2100 Bioanalyzer securing that only samples with RIN > 9 were used for subsequent sequencing applications.

### RNA sequencing and data processing

cDNA libraries were prepared from 400 to 1000 ng of total RNA using a Lexogen SENSE RNA‐Seq Kit following instructions of the manufacturer. The libraries were sequenced using a HiSeq 2500 high‐throughput flow cell. A sequencing output of 15 to 20 * 10^6^ 100‐nt‐long single reads was obtained. The complete RNAseq data set was deposited to the European Nucleotide Archive (ENA) with accession number PRJEB30287. Data quality was assessed using FastQC software. Adapter trimming was performed using the standard settings of the Cutadapt software (Martin, 2011). Quality trimming was performed using the command CLC_quality_trim from the CLC Assembly Cell software (version 5.0.1; Qiagen, Hilden, Germany). *De novo* transcriptome assembly was carried out with Trinity (Grabherr *et al*., 2011). The resulting contigs were compared with known *H. perforatum* proteins and with Arabidopsis *thaliana* (ARAPORT11) as well as *Ricinus communis* (v0.1 Phytozome) proteins using BLASTX (E‐value ≤ 10^−10^). Read mapping to the assembly was carried out with Kallisto (Bray *et al*., 2016) for each library separately. Read counts for all isoforms of a gene fragment were summed and used for calculation of differential expression using the R package DESeq2 (Love *et al*., 2014). *P*‐values were adjusted for multiple testing using the Benjamini–Hochberg method. The adopted thresholds for calling differential expression were as follows: FDR ≤ 0.01 and absolute log_2_ fold change ≥ 1.

## Authors’ contribution

P.R. conceived the idea of this study, discovered the G+/‐ PT phenotype combination, performed the characterization work, extracted RNA, interpreted transcriptomics data set and wrote the manuscript. L.A. analysed RNAseq data, identified candidate genes, assembled the novel hypericin biosynthesis pathway and contributed to the writing. P.S. performed metabolomics analysis and interpreted metabolomics results. T.R. performed confocal microscopy. A.G. established FTIR microscopy imaging methods. S.S. performed bioinformatic work. K.F. and L.W. coordinated scientific work and contributed to writing and correction of the manuscript. H.B. contributed to the correction of manuscript. M.K. provided germplasm and improved the manuscript. L.B. supervised scientific work at IPK. T.S. supervised scientific work at IPK, raised funding and contributed with corrections.

## Conflict of interest

The authors declare that they have no competing interests.

## Supporting information


**Table S1** List of characterized *H. perforatum* genotypes.
**Table S2** List of top 60 features correlating with dark glands.
**Table S3** List of top 60 features negatively correlating with dark glands.
**Table S4** Results of the statistical tests of the selected correlating features.
**Table S6** Preliminary annotation of 67 DEGs from Figure 6.
**Figure S1** Dark glands in different organs of *H. perforatum*.
**Figure S2** Currently proposed hypericin biosynthesis pathway.
**Figure S3** Distribution of dark glands per pistil in 93 genotypes of *H. perforatum*.
**Figure S4** Dissected capsules 14 days after open flower stage.
**Figure S5** FTIR analysis of placental tissue and leaf regions of *H. perforatum*.
**Figure S6** Principal component analysis of *H. perforatum* infrared spectra.
**Figure S7** PLS analysis of glanded versus glandless *Hypericum* pistils analysed by UHPLC‐ESI‐HRMS in negative mode.
**Figure S8** MS intensity boxplots of selected correlating features 1‐18 from Table 1.
**Figure S9** Scheme of comparison approaches and Venn Diagram of DEGs.
**Figure S10** Transcriptomics experimental design.
**Figure S11** Model of hypericin biosynthesis and transport based on our transcriptomics and metabolomics data.

**Table S5** Expression data of 67 DEGs shown in Figure 6 (excel file).
