# Supplementary material for: Discovery of key regulators of dark gland development and hypericin biosynthesis in St. John's Wort (Hypericum perforatum)
Source: Plant Biotechnol J. 2019 May 17;17(12):2299–312. doi: 10.1111/pbi.13141 (PMC6835128; doi:10.1111/pbi.13141)
Supplement: Supplementary file 1 — Table S1 List of characterized H. perforatum genotypes. Table S2 List of top 60 features correlating with dark glands. Table S3 List of top 60 features negatively correlating with dark glands. Table S4 Results of the statistical tests of the selected correlating features. Table S6 Preliminary annotation of 67 DEGs from Figure 6. Figure S1 Dark glands in different organs of H. perforatum. Figure S2 Currently proposed hypericin biosynthesis pathway. Figure S3 Distribution of dark glands per pistil in 93 genotypes of H. perforatum. Figure S4 Dissected capsules 14 days after open flower stage. Figure S5 FTIR analysis of placental tissue and leaf regions of H. perforatum. Figure S6 Principal component analysis of H. perforatum infrared spectra. Figure S7 PLS analysis of glanded versus glandless Hypericum pistils analysed by UHPLC‐ESI‐HRMS in negative mode. Figure S8 MS intensity boxplots of selected correlating features 1‐18 from Table 1. Figure S9 Scheme of comparison approaches and Venn Diagram of DEGs. Figure S10 Transcriptomics experimental design. Figure S11 Model of hypericin biosynthesis and transport based on our transcriptomics and metabolomics data. [file PBI-17-2299-s002.docx]

# Supplemental information

**Discovery of key regulators of dark glands development and hypericin biosynthesis in St. John’s wort (Hypericum perforatum)**

Paride Rizzo^1^, Lothar Altschmied ^1^, Pauline Stark ^2^, Twan Rutten^1^, André Guendel^1^, Sarah Scharfenberg^2^, Katrin Franke^2^, Helmut Baeumlein^1^, Ludger Wessjohann^2^, Marcus Koch^3^, Ljudmilla Borisjuk^1^ & Timothy F. Sharbel^1,4^

^1^ Leibniz-Institut für Pflanzengenetik und Kulturpflanzenforschung (IPK) – Corrensstraße 3, 06466 Gatersleben (Germany)

^2^ Leibniz-Institut für Pflanzenbiochemie (IPB) – Weinberg 3, 06120 Halle (Saale) (Germany)

^3^ Ruprecht Karls Universität Heidelberg - Im Neuenheimer Feld 345, 69120 Heidelberg (Germany)

^4^ Global Institute for Food Security (GIFS) - 110 Gymnasium Place, University of Saskatchewan Saskatoon, SK S7N 0W9 (Canada)

Corresponding author:

Paride Rizzo

Tel. +49 39 48 25 787

[rizzo@ipk-gatersleben.de](mailto:rizzo@ipk-gatersleben.de)

Content:

Table S1 – List of characterized *H. perforatum* genotypes. page 2

Table S2 – List of top 60 features correlating with dark glands. page 4

Table S3 – List of top 60 features negatively correlating with dark glands. page 6

Table S4 – Results of the statistical tests of the selected correlating features. page 8

Table S5 – Expression data of 67 DEGs shown in Figure 6 (excel file). page 8

Table S6 – Preliminary annotation of 67 DEGs from Figure 6. page 9

Figure S1 – Dark glands in different organs of *H. perforatum.* page 11

Figure S2 – Currently proposed hypericin biosynthesis pathway. page 12

Figure S3 – Distribution of dark glands per pistil in 93 genotypes of *H. perforatum*. page 12

Figure S4 – Dissected capsules 14 days after open flower stage . page 13

Figure S5 – FTIR analysis of placental tissue and leave regions of *H. perforatum.* page 14

Figure S6 – Principle component analysis of *H. perforatum* infrared spectra. page 15

Figure S7 – PLS analysis of glanded versus glandless *Hypericum* pistils analyzed by UHPLC-ESI-HRMS in negative mode. page 16

Figure S8 – MS intensity boxplots of selected correlating features 1-18 from Table 1. page 17

Figure S9 – Scheme of comparison approaches and Venn Diagram of DEGs. page 18

Figure S10 – Transcriptomics experimental design. page 19

Figure S11 – Model of hypericin biosynthesis and transport based on our transcriptomics and metabolomics data. page 20

Table S1 – H. perforatum genotypes. The number of dark glands per pistil (DGs/pistil) were obtained from five plants of each genotype from which three flowers per plant were investigated (n = 15). Ploidy was determined through flow cytometry.

| **Genotype** | **Locality (latidue / longitude)** | **Ploidy** | **DGs/pistil ± sdev** |
| --- | --- | --- | --- |
| HEID-919132 | NA | 2C | 0.0 |
| HyPR-01 | NA | 2C | 115.7 ± 8.7 |
| HyPR-02 | Iron Mountain, MI, USA (45.83 / -88.08) | 4C | 0.0 |
| HyPR-03 | NA | 2C | 95.2 ± 4.2 |
| HyPR-05 | La Selva de Mar, Spain (41.21 / 1.13) | 4C | 79.6 ± 14.2 |
| HyPR-06 | NA | 2C | 108.8 ± 16.6 |
| HyPR-08 | Tuscola, IL, USA (39.80 / -88.28) | 4C | 0.0 |
| HyPR-09 | La Selva de Mar, Spain (41.21 / 1.13) | 4C | 45.2 ± 41.1 |
| H06-1367 | Tuscola, IL, USA (39.80 / -88.28) | 4C | 0.0 |
| H06-1369 | Tuscola, IL, USA (39.80 / -88.28) | 4C | 0.0 |
| H06-1376 | Tuscola, IL, USA (39.80 / -88.28) | 4C | 0.0 |
| H06-1378 | ESGR, MI, USA (42.50 / -83.70) | 4C | 0.6 ± 1.2 |
| H06-1383 | Green Lake, WI, USA (43.85 / -89.30) | 4C | 20.2 ± 37.1 |
| H06-1406 | Green Lake, WI, USA (43.85 / -89.30) | 4C | 1.0 ± 2.2 |
| H06-1427 | Green Lake, WI, USA (43.85 / -89.30) | 4C | 0.4 ± 1.5 |
| H06-1435 | Green Lake, WI, USA (43.85 / -89.30) | 4C | 0.8 ± 1.8 |
| H06-1441 | Green Lake, WI, USA (43.85 / -89.30) | 4C | 0.0 |
| H06-1449 | Point Beach, WI, USA (44.26 / -87.56) | 6C | 77.4 ± 45.3 |
| H06-1450 | Point Beach, WI, USA (44.26 / -87.56) | 4C | 0.0 |
| H06-1453 | Point Beach, WI, USA (44.26 / -87.56) | 4C | 0.0 |
| H06-1460 | Point Beach, WI, USA (44.26 / -87.56) | 4C | 29.8 ± 46.1 |
| H06-1489 | Kewaunee, MI, USA (44.61 / -88.11) | 4C | 0.0 |
| H06-1498 | Kewaunee, MI, USA (44.61 / -88.11) | 6C | 0.0 |
| H06-1513 | Kewaunee, MI, USA (44.61 / -88.11) | 4C | 0.0 |
| H06-1533 | Kewaunee, MI, USA (44.61 / -88.11) | 6C | 6.0 ± 9.3 |
| H06-1591 | Gillett, WI, USA (44.84 / -88.61) | 4C | 1.8 ± 3.9 |
| H06-1643 | Rideau River, ON, Canada (45.00 / -75.62) | 4C | 0.0 |
| H06-1644 | Rideau River, ON, Canada (45.00 / -75.62) | 6C | 2.4 ± 5.3 |
| H06-1650 | Rideau River, ON, Canada (45.00 / -75.62) | 4C | 16.0 ± 33.5 |
| H06-1665 | Rideau River, ON, Canada (45.00 / -75.62) | 4C | 1.4 ± 3.7 |
| H06-1681 | Menominee, MI, USA (45.21 / -87.75) | 4C | 0.0 |
| H06-1698 | Menominee, MI, USA (45.21 / -87.75) | 6C | 0.0 |
| H06-1765 | Wausaukee, WI, USA (45.45 / -87.63) | 4C | 0.0 |
| H06-1819 | Carney, MI, USA (45.60 / -87.03) | 4C | 0.0 |
| H06-1823 | Carney, MI, USA (45.60 / -87.03) | 4C | 0.0 |
| H06-1877 | Carney, MI, USA (45.60 / -87.03) | 4C | 0.0 |
| H06-1883 | Iron Mountain, MI, USA (45.83 / -88.08) | 4C | 0.6 ± 1.7 |
| H06-1887 | Iron Mountain, MI, USA (45.83 / -88.08) | 6C | 3.8 ± 12.4 |
| H06-1960 | Iron Mountain, MI, USA (45.83 / -88.08) | 4C | 0.0 |
| H06-1964 | Tecumseh, MI, USA (42.00 / -89.66) | 4C | 8.2 ± 8.2 |
| H06-1979 | Tecumseh, MI, USA (42.00 / -89.66) | 4C | 83.2 ± 12.0 |
| H06-1988 | Tecumseh, MI, USA (42.00 / -89.66) | 4C | 103.2 ± 13.5 |
| H06-1994 | Cazadero, CA, USA (38.55 / -123.13) | 4C | 75.6 ± 18.9 |
| H06-1998 | Cazadero, CA, USA (38.55 / -123.13) | 4C | 64.0 ± 17.1 |
| H06-2377 | Winchester, OR, USA (43.26 / -123.36) | 4C | 0.2 ± 0.8 |
| H06-2386 | Winchester, OR, USA (43.26 / -123.36) | 4C | 0.0 |
| H06-2458 | Corvallis, OR, USA (44.61 / -123.2) | 4C | 78.0 ± 44.0 |
| H06-2464 | Granera, Spain (41.73 / 2.06) | 4C | 80.4 ±19.5 |
| H06-2755 | Bolzano, Italy (46.51 / 12.15) | 4C | 2.2 ± 3.1 |
| H06-2767 | Bolzano, Italy (46.51 / 12.15) | 4C | 4.6 ± 7.1 |
| H06-2850 | Badia Polesine, Italy (45.08 / 11.48) | 4C | 0.0 |
| H06-2857 | Badia Polesine, Italy (45.08 / 11.48) | 4C | 58.3 ± 31.1 |
| H06-2874 | Badia Polesine, Italy (45.08 / 11.48) | 4C | 0.0 |
| H06-2886 | Badia Polesine, Italy (45.08 / 11.48) | 6C | 95.9 ± 27.8 |
| H06-2887 | Badia Polesine, Italy (45.08 / 11.48) | 4C | 102.0 ± 18.1 |
| H06-2925 | Badia Polesine, Italy (45.08 / 11.48) | 4C | 6.8 ± 8.1 |
| H06-2941 | Clapier, France (44.10 / 7.41) | 4C | 52.2 ± 12.9 |
| H06-2945 | Clapier, France (44.10 / 7.41) | 4C | 38.4 ± 11.1 |
| H06-2957 | Clapier, France (44.10 / 7.41) | 4C | 37.0 ± 15.7 |
| H06-3033 | Cerbere, France (42.45 / 3.16) | 4C | 107.2 ± 12.8 |
| H06-3075 | Adliswil, Switzerland, (47.30 / 8.56) | 4C | 0.0 |
| H06-3085 | Adliswil, Switzerland, (47.30 / 8.56) | 6C | 43.2 ± 9.3 |
| H06-3087 | Adliswil, Switzerland, (47.30 / 8.56) | 4C | 47.2 ± 23.3 |
| H06-3148 | Arlesheim, Switzerland (47.48 / 7.61) | 4C | 52.0 ± 18.8 |
| H06-3154 | DeSteeg, Netherlands (52.00 / 6.05) | 4C | 102.2 ± 12.5 |
| H06-3184 | Velp, Netherlands (52.00 / 5.59) | 4C | 27.5 ± 8.8 |
| H06-3189 | Velp, Netherlands (52.00 / 5.59) | 4C | 10.0 ± 7.3 |
| H06-3190 | Velp, Netherlands (52.00 / 5.59) | 4C | 11.4 ± 15.0 |
| H06-3191 | Velp, Netherlands (52.00 / 5.59) | 4C | 0.0 |
| H06-3194 | Velp, Netherlands (52.00 / 5.59) | 4C | 1.0 ± 2.2 |
| H06-3196 | Silwood, UK (51.50 / -0.66) | 4C | 0.0 |
| H06-3197 | Silwood, UK (51.50 / -0.66) | 4C | 5.2 ± 4.6 |
| H06-3199 | Silwood, UK (51.50 / -0.66) | 4C | 0.0 |
| H06-3200 | Silwood, UK (51.50 / -0.66) | 4C | 0.0 |
| H06-3205 | Silwood, UK (51.50 / -0.66) | 4C | 2.6 ± 3.4 |
| H06-3214 | Silwood, UK (51.50 / -0.66) | 4C | 19.0 ± 14.9 |
| H06-3220 | Silwood, UK (51.50 / -0.66) | 4C | 0.0 |
| H06-3232 | Silwood, UK (51.50 / -0.66) | 4C | 0.8 ± 1.8 |
| H06-3233 | Silwood, UK (51.50 / -0.66) | 4C | 3.8 ± 5.3 |
| H06-3246 | Praha, Czech Republic (50.08 / 14.46) | 6C | 0.0 |
| H06-3251 | Praha, Czech Republic (50.08 / 14.46) | 4C | 0.0 |
| H06-3265 | Praha, Czech Republic (50.08 / 14.46) | 6C | 0.0 |
| H06-3279 | Praha, Czech Republic (50.08 / 14.46) | 4C | 0.0 |
| H06-3281 | Praha, Czech Republic (50.08 / 14.46) | 6C | 0.0 |
| H06-3286 | Praha, Czech Republic (50.08 / 14.46) | 6C | 0.0 |
| H06-3287 | Praha, Czech Republic (50.08 / 14.46) | 4C | 0.0 |
| H06-3331 | Bonn, Germany (50.73 / 7.10) | 6C | 0.0 |
| H06-3337 | Bonn, Germany (50.73 / 7.10) | 4C | 3.4 ± 4.4 |
| H06-3340 | Bonn, Germany (50.73 / 7.10) | 4C | 2.4 ± 3.8 |
| H06-3341 | Bonn, Germany (50.73 / 7.10) | 6C | 0.8 ± 1.8 |
| H06-3360 | Hamburg, Germany (52.86 / 10.58) | 4C | 0.0 |
| H06-3367 | Hamburg, Germany (52.86 / 10.58) | 4C | 0.0 |
| H06-3395 | Kewaunee, MI, USA (44.61 / -88.11) | 4C | 0.0 |

Table S2 - Data of the 60 most correlating features of the PLS model (including isotopes); sorted by retention time. M = mass, z = charge, Rt = retention time, Var1 = the variance of the feature that is explained by component 1; RDB = Ring Double Bond Equivalent

| **Feature**  **m/z/RT** | **Cor. order** | **Var1** | **Rt [min]** | **Observed *m/z*** | **Elemental composition** | **RDB** | **Δ ppm** | **Assignment** | **Ions/ Isotopes** |
| --- | --- | --- | --- | --- | --- | --- | --- | --- | --- |
| 459.1/20 | 41 | 0.897 | 0.33 | 459.18409 |  |  |  | Unknown |  |
| 337.1/172 | 42 | 0.897 | 2.87 | 337.09215 | C_16_H_17_O_8_^-^ | 8.5 | -2.197 | O-*p*-Coumaroylquinic acid | [M-H]^-^ |
| 338.1/191 | 28 | 0.912 | 3.18 | 338.09605 | C_15_ ^[13]^CH_17_O_8_^-^ | 8.5 | -0.578 | O-*p*-Coumaroylquinic acid | [M-H]^-^ +1 |
| 337.1/191 | 33 | 0.905 | 3.18 | 337.09258 | C_16_H_17_O_8_^-^ | 8.5 | -0.922 | O-*p*-Coumaroylquinic acid | [M-H]^-^ |
| 163.0/191 | 37 | 0.900 | 3.18 | 163.04034 | C_9_H_7_O_3_^-^ | 6.5 | 1.672 | O-*p*-Coumaroylquinic acid | fragment |
| 485.1/347 | 16 | 0.924 | 5.78 | 485.08508 | C_21_H_22_O_11_Cl^-^ | 10.5 | -0.768 | Unknown (449.10864) | [M+Cl]^-^ |
| 467.1/348 | 6 | 0.936 | 5.80 | 467.07392 |  |  |  | Unknown |  |
| 449.1/348 | 8 | 0.933 | 5.80 | 449.10864 | C_21_H_21_O_11_^-^ | 11.5 | -0.656 | Unknown | [M-H]^-^ |
| 509.1/348 | 27 | 0.913 | 5.80 | 509.12913 | C_23_H_25_O_13_^-^ | 11.5 | -3.445 | Unknown | [M-H]^-^ |
| 625.1/349 | 7 | 0.934 | 5.82 | 625.13918 |  |  |  | Unknown |  |
| 434.1/363 | 17 | 0.923 | 6.05 | 434.11650 | C_20_ ^[13]^CH_21_O_10_^-^ | 11.5 | -2.015 | Unknown (433.11305) | [M-H]^-^ +1 |
| 467.1/367 | 9 | 0.933 | 6.12 | 467.11199 | C_19_ ^[13]^C_2_H_21_O_12_^-^ | 11.5 | 3.064 | Unknown (465.10358) | [M-H]^-^ +2 |
| 307.1/368 | 34 | 0.904 | 6.13 | 307.13942 | C_13_H_23_O_8_^-^ | 2.5 | -2.477 | Unknown | [M-H]^-^ |
| 308.1/368 | 55 | 0.890 | 6.13 | 308.14242 | C_12_ ^[13]^CH_23_O_8_^-^ | 2.5 | -2.517 | Unknown (307.13942) | [M-H]^-^ +1 |
| 419.1/398 | 31 | 0.909 | 6.63 | 419.09722 | C_20_H_19_O_10_^-^ | 11.5 | 2.744 | Unknown |  |
| 896.2/425 | 21 | 0.918 | 7.08 | 896.19602 | C_41_ ^[13]^CH_39_O_22_^-^ | 23.5 | -1.317 | Quercetin-3-*O*-rhamnosid | [2M-H]^-^ +1 |
| 895.2/425 | 30 | 0.909 | 7.08 | 895.19177 | C_42_H_39_O_22_^-^ | 23.5 | -2.319 | Quercetin-3-*O*-rhamnosid | [2M-H]^-^ |
| 897.2/425 | 43 | 0.896 | 7.08 | 897.19961 | C_40_ ^[13]^C_2_H_39_O_22_^-^ | 23.5 | -1.054 | Quercetin-3-*O*-rhamnosid | [2M-H]^-^ +2 |
| 583.1/425 | 45 | 0.894 | 7.08 | 583.06813 |  |  |  | Unknown |  |
| 577.1/425 | 48 | 0.894 | 7.08 | 577.05081 |  |  |  | Unknown |  |
| 447.1/425 | 51 | 0.892 | 7.08 | 447.09218 | C_21_H_19_O_11_^-^ | 12.5 | -2.471 | Quercetin-3-*O*-rhamnosid | [M-H]^-^ |
| 515.1/425 | 52 | 0.892 | 7.08 | 515.07949 |  |  |  | Unknown |  |
| 532.1/426 | 29 | 0.910 | 7.10 | 532.06560 |  |  |  | Unknown (531.05338) | [M-H]^-^ +1 |
| 463.1/445 | 44 | 0.895 | 7.42 | 463.08772 | C_21_H_19_O_12_^-^ | 12.5 | -1.035 | Quercetin-glucosid | [M-H]^-^ |
| 494.1/455 | 19 | 0.919 | 7.58 | 494.09320 |  |  |  | Unknown |  |
| 499.1/455 | 26 | 0.914 | 7.58 | 499.08554 |  |  |  | Unknown |  |
| 603.1/461 | 36 | 0.901 | 7.68 | 603.07681 | C_30_H_19_O_14_^-^ | 21.5 | -2.020 | Quercetin | [2M-H]^-^ |
| 604.1/461 | 38 | 0.899 | 7.68 | 604.08050 | C_29_ ^[13]^CH_19_O_14_^-^ | 21.5 | -1.462 | Quercetin | [2M-H]^-^ +1 |
| 304.0/461 | 46 | 0.894 | 7.68 | 304.04262 | C_12_ ^[13]^C_3_H_9_O_7_^-^ | 11.5 | -9.276 | Quercetin | [M-H]^-^ +3 |
| 301.0/461 | 54 | 0.891 | 7.68 | 301.03534 | C_15_H_9_O_7_^-^ | 11.5 | -0.119 | Quercetin | [M-H]^-^ |
| 302.0/461 | 58 | 0.889 | 7.68 | 302.03858 | C_14_ ^[13]^CH_9_O_7_^-^ | 11.5 | 0.499 | Quercetin | [M-H]^-^ +1 |
| 313.0/501 | 10 | 0.931 | 8.35 | 313.03481 | C_16_H_9_O_7_^-^ | 12.5 | -1.808 | Endocrocin | [M-H]^-^ |
| 525.1/599 | 2 | 0.941 | 9.98 | 525.11854 | C_30_H_21_O_9_^-^ | 20.5 | -1.077 | Hydroxyemodin dianthrone  or Hydroxypenicilliopsin | [M-H]^-^ |
| 526.1/599 | 5 | 0.937 | 9.98 | 526.12088 | C_29_ ^[13]^CH_21_O_9_^-^ | 20.5 | -3.004 | Hydroxyemodin dianthrone  or Hydroxypenicilliopsin | [M-H]^-^ +1 |
| 699.1/664 | 15 | 0.924 | 11.07 | 699.13538 | C_36_H_27_O_15_^-^ | 23.5 | -0.233 | S-(+)-Skyrin-6-O-β-glucopyranoside | [M-H]^-^ |
| 701.1/664 | 24 | 0.917 | 11.07 | 701.13997 | C_34_ ^[13]^C_2_H_27_O_15_^-^ | 23.5 | -3.256 | S-(+)-Skyrin-6-O-β-glucopyranoside | [M-H]^-^ +2 |
| 700.1/664 | 25 | 0.915 | 11.07 | 700.13780 | C_35_ ^[13]^CH_27_O_15_^-^ | 23.5 | -1.568 | S-(+)-Skyrin-6-O-β-glucopyranoside | [M-H]^-^ +1 |
| 670.1/669 | 1 | 0.948 | 11.15 | 670.12798 | C_34_ ^[13]^CH_25_O_14_^-^ | 23.5 | -0.527 | S-(+)-Skyrin-6-O-β-arabinofuranoside | [M-H]^-^ +1 |
| 669.1/669 | 18 | 0.923 | 11.15 | 669.12499 | C_35_H_25_O_14_^-^ | 23.5 | 0.017 | S-(+)-Skyrin-6-O-β-arabinofuranoside | [M-H]^-^ |
| 509.1/684 | 35 | 0.904 | 11.40 | 509.12402 | C_30_H_21_O_8_^-^ | 20.5 | -0.336 | Emodin dianthrone  or Penicilliopsin | [M-H]^-^ |
| 519.1/698 | 3 | 0.939 | 11.63 | 519.07155 | C_30_H_15_O_9_^-^ | 23.5 | -1.166 | Pseudohypericin | [M-H]^-^ |
| 522.1/698 | 11 | 0.930 | 11.63 | 522.08179 | C_27_ ^[13]^C_3_H_15_O_9_^-^ | 23.5 | -0.823 | Pseudohypericin | [M-H]^-^ +3 |
| 521.1/698 | 14 | 0.925 | 11.63 | 521.07853 | C_28_ ^[13]^C_2_H_15_O_9_^-^ | 23.5 | -0.643 | Pseudohypericin | [M-H]^-^ +2 |
| 485.3/764 | 23 | 0.917 | 12.73 | 485.32702 | C_30_H_45_O_5_^-^ | 8.5 | -0.469 | Unknown | [M-H]^-^ |
| 520.1/768 | 39 | 0.898 | 12.80 | 520.07505 | C_29_ ^[13]^CH_15_O_9_^-^ | 23.5 | -0.885 | Pseudohypericin,  peak tailing | [M-H]^-^ +1 |
| 519.1/768 | 49 | 0.893 | 12.80 | 519.07189 | C_30_H_15_O_9_^-^ | 23.5 | -0.511 | Pseudohypericin,  peak tailing | [M-H]^-^ |
| 506.1/776 | 4 | 0.938 | 12.93 | 506.09603 | C_29_ ^[13]^CH_17_O_8_^-^ | 22.5 | -0.426 | Protohypericin | [M-H]^-^ +1 |
| 505.1/776 | 13 | 0.928 | 12.93 | 505.0929 | C_30_H_17_O_8_^-^ | 22.5 | 0.018 | Protohypericin | [M-H]^-^ |
| 473.3/794 | 47 | 0.894 | 13.23 | 473.31801 | C_27_ ^[13]^C_2_H_43_O_5_^-^ | 8.5 | -0.628 | Unknown (471.31093) | [M-H]^-^ +2 |
| 471.3/794 | 59 | 0.889 | 13.23 | 471.31093 | C_29_H_43_O_5_^-^ | 8.5 | -1.417 | Unknown | [M-H]^-^ |
| 472.3/794 | 60 | 0.887 | 13.23 | 472.31461 | C_28_ ^[13]^CH^43^O_5_^-^ | 8.5 | -0.725 | Unknown (471.31093) | [M-H]^-^ +1 |
| 540.4/795 | 32 | 0.908 | 13.25 | 540.37734 | C_33_ ^[13]^CH_51_O_5_^-^ | 9.5 | -0.394 | Unknown (539.37403) | [M-H]^-^ +1 |
| 539.4/795 | 40 | 0.898 | 13.25 | 539.37403 | C_34_H_51_O_5_^-^ | 9.5 | -0.311 | Unknown | [M-H]^-^ |
| 505.1/819 | 12 | 0.929 | 13.65 | 505.08362 | C_28_ ^[13]^C_2_H_15_O_8_^-^ | 23.5 | -0.654 | Hypericin | [M-H]^-^ +2 |
| 503.1/820 | 22 | 0.917 | 13.67 | 503.07679 | C_30_H_15_O_8_^-^ | 23.5 | -0.896 | Hypericin | [M-H]^-^ |
| 485.4/859 | 57 | 0.889 | 14.32 | 485.39901 | C_32_H_53_O_3_^-^ | 6.5 | -2.078 | Unknown |  |
| 499.4/867 | 20 | 0.918 | 14.45 | 499.41458 | C_33_H_55_O_3_^-^ | 6.5 | -2.18 | Tetracosyl *p*-coumarate | [M-H]^-^ |
| 500.4/867 | 50 | 0.893 | 14.45 | 500.41890 | C_32_ ^[13]^CH_55_O_3_^-^ | 6.5 | -0.247 | Tetracosyl *p*-coumarate (499.41458) | [M-H]^-^ +1 |
| 501.4/867 | 56 | 0.890 | 14.45 | 501.42120 | C_31_ ^[13]^C_2_H_55_O_3_^-^ | 6.5 | -2.350 | Tetracosyl *p-*coumarate (499.41458) | [M-H]^-^ +2 |
| 513.4/875 | 53 | 0.892 | 14.58 | 513.43034 | C_34_H_57_O_3_^-^ | 6.5 | -1.907 | Unknown | [M-H]^-^ |

Table S3 - Data of the 60 most negative correlating features of the PLS model; sorted by retention time. M = mass, z = charge, Rt = retention time, Var1 = the variance of the feature that is explained by component 1; RDB = Ring Double Bond Equivalent

| Feature  m/z/RT | Cor. order | Var1 | Rt [min] | Observed *m/z* | Elemental composition | RDB | Δ ppm | Assignment | Ions/ Isotopes |
| --- | --- | --- | --- | --- | --- | --- | --- | --- | --- |
| 313.1/20 | 58 | -0.748 | 0.33 | 313.11407 | C_11_H_21_O_10_^-^ | 1,5 | 0,160 | Unknown | [M-H]^-^ |
| 306.1/177 | 16 | -0.829 | 2.95 | 306.06981 | C_14_ ^[13]^CH_13_O_7_^-^ | 9.5 | -0,721 | Unknown (305.06634) | [M-H]^-^ +1 |
| 305.1/177 | 19 | -0.820 | 2.95 | 305.06634 | C_15_H_13_O_7_^-^ | 9.5 | 2.494 | Unknown | [M-H]^-^ |
| 289.1/344 | 22 | -0.816 | 5.73 | 289.12927 | C_13_H_21_O_7_^-^ | 3.5 | -0.022 | Unknown | [M-H]^-^ |
| 509.2/380 | 36 | -0.798 | 6.33 | 509.22378 | C_22_H_37_O_13_^-^ | 4.5 | -0.362 | Unknown | [M-H]^-^ |
| 371.1/388 | 26 | -0.807 | 6.47 | 371.13350 | C_17_H_23_O_9_^-^ | 6.5 | -3.383 | Unknown | [M-H]^-^ |
| 609.1/399 | 46 | -0.779 | 6.65 | 609.14549 | C_27_H_29_O_15_^-^ | 13.5 | -1.014 | Rutin | [M-H]^-^ |
| 401.1/423 | 49 | -0.772 | 7.05 | 401.08771 | C_20_H_17_O_9_^-^ | 12.5 | -0.238 | Unknown | [M-H]^-^ |
| 371.1/427 | 3 | -0.899 | 7.12 | 371.13449 | C_17_H_23_O_9_^-^ | 6.5 | -0.715 | Unknown | [M-H]^-^ |
| 409.2/458 | 38 | -0.796 | 7.63 | 409.20765 | C_18_H_33_O_10_^-^ | 2.5 | -0.661 | Unknown | [M-H]^-^ |
| 631.3/460 | 35 | -0.802 | 7.67 | 631.25799 | C_29_H_43_O_15_^-^ | 8.5 | -4.362 | Unknown | [M-H]^-^ |
| 263.1/462 | 17 | -0.827 | 7.70 | 263.12801 | C_15_H_19_O_4_^-^ | 6.5 | -3.315 | Unknown | [M-H]^-^ |
| 385.1/464 | 23 | -0.812 | 7.73 | 385.11285 | C_17_H_21_O_10_^-^ | 7.5 | -3.038 | Unknown | [M-H]^-^ |
| 415.2/516 | 6 | -0.871 | 8.60 | 415.17211 | C_23_H_27_O_7_^-^ | 10.5 | -7.273 | Unknown | [M-H]^-^ |
| 467.2/621 | 34 | -0.802 | 10.35 | 467.16523 | C_33_H_23_O_3_^-^ | 22.5 | 2.267 | Unknown | [M-H]^-^ |
| 331.2/621 | 48 | -0.776 | 10.35 | 331.19116 | C_20_H_27_O_4_^-^ | 7.5 | 2.338 | Unknown | [M-H]^-^ |
| 532.3/650 | 30 | -0.807 | 10.83 | 531.33208 | C_31_H_47_O_7_^-^ | 8.5 | -1.218 | Unknown (531.33369) | [M-H]^-^ +1 |
| 546.3/665 | 51 | -0.769 | 11.08 | 546.31502 | C_30_ ^[13]^CH_45_O_8_^-^ | 9.5 | -0,597 | Unknown (545.31032) | [M-H]^-^ +1 |
| 545.3/665 | 52 | -0.768 | 11.08 | 545.31032 | C_31_H_45_O_8_^-^ | 9.5 | -1.054 | Unknown | [M-H]^-^ |
| 529.3/668 | 21 | -0.818 | 11.13 | 529.31582 | C_31_H_45_O_7_^-^ | 9.5 | -2.375 | Unknown | [M-H]^-^ |
| 513.3/674 | 53 | -0.768 | 11.23 | 513.32029 | C_31_H_45_O_6_^-^ | 9.5 | -3.648 | Unknown | [M-H]^-^ |
| 488.3/683 | 31 | -0.805 | 11.38 | 488.30886 |  |  |  | Unknown |  |
| 461.3/699 | 44 | -0.785 | 11.65 | 461.25438 | C_26_H_37_O_7_^-^ | 8.5 | 2.168 | Unknown (461.25438) | [M-H]^-^ |
| 462.3/699 | 57 | -0.752 | 11.65 | 462.25092 | C_25_ ^[13]^CH_37_O_7_^-^ | 8.5 | -14,952 | Unknown | [M-H]^-^ +1 |
| 445.3/702 | 37 | -0.797 | 11.70 | 445.25943 | C_26_H_37_O_6_^-^ | 8.5 | 2.167 | Unknown | [M-H]^-^ |
| 497.3/709 | 4 | -0.887 | 11.82 | 497.32733 | C_31_H_45_O_5_^-^ | 9.5 | 2.371 | Unknown | [M-H]^-^ |
| 498.3/709 | 27 | -0.872 | 11.82 | 498.33048 | C_30_ ^[13]^CH_45_O_5_^-^ | 9.5 | -0,246 | Unknown (497.32733) | [M-H]^-^ +1 |
| 529.3/709 | 56 | -0.760 | 11.82 | 529.31516 | C_31_H_45_O_7_^-^ | 9.5 | -3.622 | Unknown | [M-H]^-^ |
| 446.3/712 | 29 | -0.809 | 11.87 | 446.26352 | C_25_ ^[13]^CH_37_O_6_^-^ | 8.5 | 1.351 | Unknown (445.25951) | [M-H]^-^ +1 |
| 445.3/712 | 32 | -0.803 | 11.87 | 445.25951 | C_26_H_37_O_6_^-^ | 8.5 | 2.346 | Unknown | [M-H]^-^ |
| 496.3/718 | 40 | -0.790 | 11.97 | 496.31497 | C_30_ ^[13]^CH_43_O_5_^-^ | 10.5 | 0.035 | Unknown (495.31156) | [M-H]^-^ +1 |
| 429.3/718 | 50 | -0.771 | 11.97 | 429.26517 | C_26_H_37_O_5_^-^ | 8.5 | 1.217 | Unknown | [M-H]^-^ |
| 346.2/721 | 33 | -0.806 | 12.02 | 346.21035 | C_20_ ^[13]^CH_29_O_4_^-^ | 7.5 | -0,397 | Unknown (345.20693) | [M-H]^-^ +1 |
| 345.2/721 | 47 | -0.779 | 12.02 | 345.20693 | C_21_H_29_O_4_^-^ | 7.5 | 2.590 | Unknown | [M-H]^-^ |
| 331.2/727 | 1 | -0.925 | 12.12 | 331.19105 | C_20_H_27_O_4_^-^ | 7.5 | -1.306 | Phloroglucinol derivative | [M-H]^-^ |
| 441.3/733 | 39 | -0.795 | 12.22 | 441.26337 | C_27_H_37_O_5_^-^ | 9.5 | -2.895 | Unknown | [M-H]^-^ |
| 482.3/758 | 12 | -0.844 | 12.63 | 482.33522 | C_30_ ^[13]^CH_45_O_4_^-^ | 9.5 | -0.970 | Unknown (481.33224) | [M-H]^-^ +1 |
| 481.3/758 | 13 | -0.841 | 12.63 | 481.33224 | C_31_H_45_O_4_^-^ | 9.5 | 2.085 | Unknown | [M-H]^-^ |
| 497.3/758 | 41 | -0.790 | 12.63 | 497.32725 | C_31_H_45_O_5_^-^ | 9.5 | 2.210 | Unknown | [M-H]^-^ |
| 498.3/758 | 42 | -0.787 | 12.63 | 498.33006 | C_30_ ^[13]^CH_45_O_5_^-^ | 9.5 | -1.089 | Unknown (497.32725) | [M-H]^-^ +1 |
| 499.3/759 | 55 | -0.763 | 12.65 | 499.33526 | C_29_ ^[13]^C_2_H_45_O_5_^-^ | 9.5 | 2,609 | Unknown (497.32725) | [M-H]^-^ +2 |
| 549.3/761 | 9 | -0.859 | 12.68 | 549.31720 | C_41_H_41_O^-^ | 21.5 | 3.655 | Unknown | [M-H]^-^ |
| 483.3/761 | 18 | -0.823 | 12.68 | 483.33956 | C_29_ ^[13]^C_2_H_45_O_4_^-^ | 9,5 | 1.070 | Unknown (481.33163) | [M-H]^-^ +2 |
| 484.3/761 | 43 | -0.786 | 12.68 | 484.34264 | C_28_ ^[13]^C_3_H_45_O_4_^-^ | 9,5 | 0.501 | Unknown (481.33163) | [M-H]^-^ +3 |
| 550.3/761 | 59 | -0.748 | 12.68 | 550.31950 | C_40_ ^[13]^CH_41_O^-^ | 21.5 | -0,262 | Unknown (413.22944) | [M-H]^-^ +1 |
| 415.3/765 | 25 | -0.808 | 12.75 | 415.23979 | C_23_ ^[13]^C_2_H_33_O_5_^-^ | 9.5 | -0.643 | Unknown (413.22944) | [M-H]^-^ +2 |
| 414.3/767 | 15 | -0.831 | 12.78 | 414.23547 | C_24_ ^[13]^CH_33_O_5_^-^ | 9.5 | -2,975 | Unknown (413.22944) | [M-H]^-^ +1 |
| 497.4/769 | 10 | -0.856 | 12.82 | 495.34923 | C_32_H_47_O_4_^-^ | 9.5 | 4.731 | Unknown | [M-H]^-^ |
| 443.3/770 | 24 | -0.811 | 12.83 | 443.28080 | C_27_H_39_O_5_^-^ | 8.5 | 3.608 | Unknown | [M-H]^-^ |
| 403.3/793 | 5 | -0.877 | 13.22 | 403.27509 | C_23_ ^[13]^C_2_H_37_O_4_^-^ | 7.5 | -3.627 | Unknown (401.26953) | [M-H]^-^ +2 |
| 442.3/793 | 11 | -0.850 | 13.22 | 442.30374 | C_27_ ^[13]^CH_41_O_4_^-^ | 8,5 | -1,465 | Unknown (441.30070) | [M-H]^-^ +1 |
| 441.3/793 | 20 | -0.819 | 13.22 | 441.30070 | C_28_H_41_O_4_^-^ | 8.5 | -0.755 | Unknown | [M-H]^-^ |
| 402.3/793 | 28 | -0.807 | 13.22 | 402.27289 | C_24_ ^[13]^CH_37_O_4_^-^ | 7.5 | -0.491 | Unknown (401.26953) | [M-H]^-^ +1 |
| 498.3/795 | 60 | -0.747 | 13.25 | 498.33128 | C_30_ ^[13]^CH_45_O_5_^-^ | 9,5 | 1,359 | Unknown | [M-H]^-^ +1 |
| 497.3/796 | 54 | -0.764 | 13.27 | 497.32714 | C_31_H_45_O_5_^-^ | 9.5 | -0.217 | Unknown | [M-H]^-^ |
| 617.5/812 | 7 | -0.867 | 13.53 | 617.45675 | C_41_H_61_O_4_^-^ | 11.5 | 0.507 | Unknown | [M-H]^-^ |
| 618.5/812 | 8 | -0.860 | 13.53 | 618.46124 | C_40_ ^[13]^CH_61_O_4_^-^ | 11.5 | 0,568 | Unknown | [M-H]^-^ +1 |
| 822.5/835 | 14 | -0.836 | 13.92 | 822.53266 | C_52_ ^[13]^CH_73_ O_7_^-^ | 17.5 | -8,355 | Unknown (821.53548) | [M-H]^-^ +1 |
| 821.5/835 | 45 | -0.784 | 13.92 | 821.53548 | C_53_H_73_O_7_^-^ | 17,5 | -0,849 | Unknown | [M-H]^-^ |
| 619.5/886 | 2 | -0.913 | 14.77 | 619.47200 | C_41_H_63_O_4_^-^ | 10,5 | -1,911 | Unknown | [M-H]^-^ +1 |

Table S4 - Results of the statistical tests of the selected correlating features of the PLS model (Table 1). Table sorted by correlation order with decreasing correlation factor. Analysis of variance conducted using the F-Test (p-value > 0.05 hypothesis rejected) with homogeneous variances in black and nonhomogeneous in green. In case of homoscedasticity the t-Test was performed, in case of heteroscedasticity the variance independent Welch two sample t-Test. Effect size defined by Cohen and power calculation was done feature wise with the pooled variance.

| **Number** | **Correlation order** | **Feature** | **p-value**  **F-Test** | **p-value**  **t-Test** | **p-value WelchTest** | **EffectSize** | **Power** |
| --- | --- | --- | --- | --- | --- | --- | --- |
| 1 | 2 | 525.1/599 | 1.14E-05 | 3.01E-07 | 2.44E-05 | 3.951 | 1 |
| 2 | 3 | 519.1/698 | 8.35E-04 | 1.95E-07 | 1.14E-05 | 4.082 | 1 |
| 3 | 8 | 449.1/348 | 5.70E-03 | 3.81E-08 | 2.34E-06 | 4.604 | 1 |
| 4 | 10 | 313/501 | 1.71E-05 | 6.44E-08 | 9.90E-06 | 4.432 | 1 |
| 5 | 13 | 505.1/776 | 1.24E-04 | 7.71E-08 | 8.96E-06 | 4.374 | 1 |
| 6 | 15 | 699.1/664 | 7.68E-06 | 1.79E-06 | 6.98E-05 | 3.439 | 1 |
| 7 | 18 | 669.1/669 | 1.75E-03 | 1.12E-07 | 6.81E-06 | 4.255 | 1 |
| 8 | 22 | 503.1/820 | 1.77E-03 | 1.15E-08 | 1.67E-06 | 5.015 | 1 |
| 9 | 26 | 499.1/455 | 5.32E-05 | 8.66E-08 | 1.06E-05 | 4.336 | 1 |
| 10 | 29 | 532.1/426 | 2.44E-01 | 3.42E-05 | 6.14E-05 | 2.677 | 1 |
| 11 | 33 | 337.1/191 | 4.45E-01 | 4.30E-05 | 5.59E-05 | 2.622 | 0.999 |
| 12 | 35 | 509.1/684 | 1.62E-04 | 2.25E-09 | 1.13E-06 | 5.623 | 1 |
| 13 | 42 | 337.1/172 | 5.76E-01 | 9.47E-06 | 1.14E-05 | 2.998 | 1 |
| 14 | 44 | 463.1/445 | 1.29E-01 | 6.26E-08 | 4.42E-07 | 4.441 | 1 |
| 15 | 51 | 447.1/425 | 6.99E-01 | 3.48E-06 | 3.87E-06 | 3.260 | 1 |
| 16 | 54 | 301/461 | 6.99E-02 | 8.74E-09 | 1.69E-07 | 5.114 | 1 |
|  |  |  |  |  |  |  |  |
| 18 | -46 | 609.1/399 | 2.479E-03 | 3.012E-04 | 1.167E-03 | 2.164 | 0.990 |
|  |  |  |  |  |  |  |  |

*Table S5 – Expression data of 67 DEGs shown in Figure 6. Log2 fold changes (log2FC), adjusted P values (padj) for all comparisons given in Figure S9 and read counts as well as normalized read counts for all samples. Differentially expressed genes (padj ≤ 0.01 and | lg2FC | ≥ 1) in any comparison are marked with colours as in Figure S9. See Excel file: “****TableS5.xlsx****”.*

Table S6 – Preliminary annotation of 67 differentially expressed gene fragments for which the expression profile is shown in the six k-means clusters of Figure 6. Gene fragments obtained by TRINITY assembly of RNA-seq reads were compared (BLASTX) with protein sequences from H. perforatum (bold), Arabidopsis thaliana (ARAPORT11), if no hit was observed with H. perforatum proteins, and Ricinus communis (v0.1, Phytozome; italics), if no hit was observed with A. thaliana proteins. NAs indicate that no BLASTX hit with any of the above proteins was found with an E-value below 1e-10. Colours refer to putative functions in hypericin biosynthesis and its regulation based on the annotation of BLASTX matches (yellow: developmental regulation; violet: biosynthetic processes associated with dark glands; green: metabolite transport; white: unknown or not attempted for clusters 4-6). General features observed in proteins are indicated as follows: transcription factor (TF); trans-membrane protein (TM); signal peptide for protein export (SIG). Abbreviations for the proposed functions are: 2-oxoglutarate and Fe(II)-dependent dioxygenase (2-ODD); ABC transporter (ABC); agamous-like 6 transcription factor (AGL6); berberine bridge enzyme (BBE); ß-glucosidase (BGLU); cytochrome P450 (CYP); glutathione S-transferase (GST); major facilitator protein (MFP); MYB38 transcription factor (MYB38); octaketide synthase (OKS); polyketide cyclase (PKC); phenolic oxidative coupling protein (POCP); thioesterase (TER).

| Cluster | TRINITY ID | Gene ID | E-value | Description | Feature | Function |
| --- | --- | --- | --- | --- | --- | --- |
| 1 | DN50896_c0_g1 | AT2G45650.1 | 8e-70 | AGAMOUS-like 6 | TF | AGL6 |
| 1 | DN49520_c0_g1 | AT2G36890.2 | 6e-72 | duplicated homeodomain-like superfamily protein | TF | MYB38 |
| 1 | DN55022_c0_g1 | AT1G06620.1 | 2e-119 | 2-oxoglutarate and Fe(II)-dependent oxygenase superfamily protein |  | 2-ODD |
| 1 | DN62219_c0_g1 | AT5G17030.1 | 6e-86 | UDP-glucosyl transferase 78D3 |  | UGT |
| 1 | DN50181_c0_g1 | AT1G15520.1 | 2e-172 | pleiotropic drug resistance 12 | TM | ABC |
| 1 | DN30170_c0_g1 | AT1G10370.1 | 6e-40 | glutathione S-transferase family protein |  | GST |
| 1 | DN51158_c0_g1 | AT5G17220.1 | 9e-91 | glutathione S-transferase phi 12 |  | GST |
| 1 | DN56162_c1_g1 | AT1G10370.1 | 3e-88 | glutathione S-transferase family protein |  | GST |
| 1 | DN56162_c1_g2 | AT1G10360.1 | 2e-52 | glutathione S-transferase TAU 18 |  | GST |
| 1 | DN54001_c0_g2 | AT1G15125.1 | 5e-30 | S-adenosyl-L-methionine-dependent methyltransferases superfamily protein |  |  |
|  |  |  |  |  |  |  |
| **2** | **DN43146_c0_g1** | **EF186676.1** | **2e-25** | **octaketide synthase** |  | **OKS** |
| **2** | **DN49380_c0_g2** | **HQ529467.1** | **2e-83** | **octaketide synthase** |  | **OKS** |
| **2** | **DN55320_c0_g2** | **EU635882.1** | **0** | **octaketide synthase** |  | **OKS** |
| **2** | **DN49497_c0_g1** | **KU744669.1** | **2e-110** | **phenolic oxidative coupling protein** |  | **POCP** |
| **2** | **DN53638_c0_g1** | **KU744670.1** | **4e -12** | **phenolic oxidative coupling protein** |  | **POCP** |
| **2** | **DN53638_c0_g2** | **KU744670.1** | **3e-90** | **phenolic oxidative coupling protein** |  | **POCP** |
| **2** | **DN53898_c0_g1** | **KU744671.1** | **3e-109** | **phenolic oxidative coupling protein** |  | **POCP** |
| **2** | **DN58298_c0_g1** | **MF095120.1** | **2e-114** | **pathogenesis related class 10 protein** |  | **POCP** |
| *2* | *DN50632_c0_g2* | *29726.m004110* | *1e-36* | *major allergen Pru ar, putative* |  | *POCP* |
| 2 | DN50689_c0_g1 | AT4G24380.1 | 1e-95 | dihydrofolate reductase |  | TER |
| 2 | DN55302_c0_g1 | AT2G44480.1 | 0 | beta glucosidase 17 | SIG | BGLU |
| 2 | DN58399_c0_g1 | AT4G20800.1 | 0 | FAD-binding Berberine family protein | SIG | BBE |
| 2 | DN58309_c1_g3 | AT1G10360.1 | 2e-53 | glutathione S-transferase TAU 18 |  | GST |
|  |  |  |  |  |  |  |
| 3 | DN47561_c0_g1 | AT4G01650.1 | 3e-34 | polyketide cyclase / dehydrase and lipid transport protein |  | PKC |
| 3 | DN36476_c0_g1 | AT4G24380.1 | 2e-56 | dihydrofolate reductase |  | TER |
| 3 | DN56533_c0_g1 | AT5G06900.1 | 3e-125 | cytochrome P450, family 93, subfamily D, polypeptide 1 | TM | CYP |
| 3 | DN48718_c0_g1 | AT3G53480.1 | 0 | pleiotropic drug resistance 9 | TM | ABC |
| 3 | DN49538_c0_g1 | AT1G15520.1 | 1e-74 | pleiotropic drug resistance 12 | TM | ABC |
| 3 | DN49999_c0_g1 | AT1G15520.1 | 9e-134 | pleiotropic drug resistance 12 | TM | ABC |
| 3 | DN56439_c0_g3 | AT1G54730.2 | 1e-109 | major facilitator superfamily protein | TM | MFP |
| *3* | *DN46947_c0_g1* | *29736.m002065* | *3e-12* | *glutathione S-transferase, putative* |  | *GST* |
| 3 | DN33603_c0_g1 | AT4G11650.1 | 1e-82 | osmotin 34 |  |  |
| 3 | DN42696_c0_g1 | AT1G14730.1 | 3e-49 | cytochrome b561/ferric reductase transmembrane protein family | TM |  |
|  |  |  |  |  |  |  |
| 4 | DN11213_c0_g1 | NA | NA | NA |  |  |
| 4 | DN23784_c0_g1 | AT3G57030.1 | 2e-50 | calcium-dependent phosphotriesterase superfamily protein |  |  |
| 4 | DN27968_c0_g1 | AT3G15270.1 | 5e-20 | squamosa promoter binding protein-like 5 |  |  |
| *4* | *DN33389_c0_g1* | *30131.m007102* | *1e-18* | *conserved hypothetical protein* |  |  |
| 4 | DN34061_c0_g1 | AT5G62460.1 | 2e-28 | RING/FYVE/PHD zinc finger superfamily protein |  |  |
| *4* | *DN37948_c0_g1* | *30174.m008936* | *2e-28* | *MADS-box protein, putative* |  |  |
| 4 | DN39369_c0_g1 | NA | NA | NA |  |  |
| 4 | DN42973_c0_g1 | AT5G61280.1 | 3e-27 | remorin family protein |  |  |
| 4 | DN45016_c0_g1 | AT5G64667.1 | 2e-11 | inflorescence deficient in abscission (IDA)-like 2 |  |  |
| 4 | DN46744_c0_g1 | AT5G58140.1 | 0 | phototropin 2 |  |  |
| *4* | *DN49570_c0_g1* | *30020.m000201* | *2e-20* | *conserved hypothetical protein* |  |  |
| 4 | DN50273_c0_g2 | NA | NA | NA |  |  |
| 4 | DN50643_c0_g1 | NA | NA | NA |  |  |
| 4 | DN51004_c0_g1 | AT1G69120.1 | 6e-108 | K-box region and MADS-box transcription factor family protein |  |  |
| 4 | DN51571_c0_g1 | AT5G58140.1 | 0 | phototropin 2 |  |  |
| 4 | DN52062_c0_g1 | NA | NA | NA |  |  |
| 4 | DN53836_c0_g1 | AT4G30470.1 | 1e-119 | NAD(P)-binding Rossmann-fold superfamily protein |  |  |
| 4 | DN54008_c0_g1 | AT1G52565.1 | 8e-14 | cytochrome P450 family protein |  |  |
| 4 | DN55134_c1_g1 | AT4G03230.1 | 3e-128 | G-type lectin S-receptor-like Serine/Threonine-kinase |  |  |
| 4 | DN56322_c1_g1 | AT3G27200.1 | 2e-13 | cupredoxin superfamily protein |  |  |
| 4 | DN57073_c0_g1 | AT4G23420.1 | 2e-159 | NAD(P)-binding Rossmann-fold superfamily protein |  |  |
| 4 | DN57553_c0_g1 | AT3G44190.1 | 3e-152 | FAD/NAD(P)-binding oxidoreductase family protein |  |  |
| 4 | DN62894_c0_g2 | NA | NA | NA |  |  |
| 4 | DN63398_c1_g1 | AT5G64300.1 | 0 | GTP cyclohydrolase II |  |  |
|  |  |  |  |  |  |  |
| 5 | DN46978_c0_g1 | AT3G48660.1 | 9e-35 | transmembrane protein, putative (DUF 3339) |  |  |
| 5 | DN54519_c1_g1 | AT2G21600.1 | 5e-95 | endoplasmatic reticulum retrieval protein 1B |  |  |
| 5 | DN58039_c0_g2 | AT1G30040.1 | 1e-160 | gibberellin 2-oxidase |  |  |
| 5 | DN59540_c0_g1 | AT5G13870.1 | 0 | xyloglucan endotransglucosylase/hydrolase 5 |  |  |
| 5 | DN60595_c1_g1 | AT3G11340.1 | 2e-135 | UDP-glycosyltransferase superfamily protein |  |  |
|  |  |  |  |  |  |  |
| *6* | *DN46330_c0_g1* | *29816.m000674* | *9e-11* | *conserved hypothetical protein* |  |  |
| 6 | DN48219_c0_g1 | AT1G13580.1 | 6e-87 | LAG1 longevity assurance-like protein |  |  |
| 6 | DN48590_c0_g1 | AT4G15093.1 | 4e-107 | catalytic LigB subunit of aromatic ring-opening dioxygenase family |  |  |
| 6 | DN53327_c0_g1 | AT1G05000.1 | 3e-109 | phosphotyrosine protein phosphatases superfamily protein |  |  |
| 6 | DN55007_c0_g1 | NA | NA | NA |  |  |


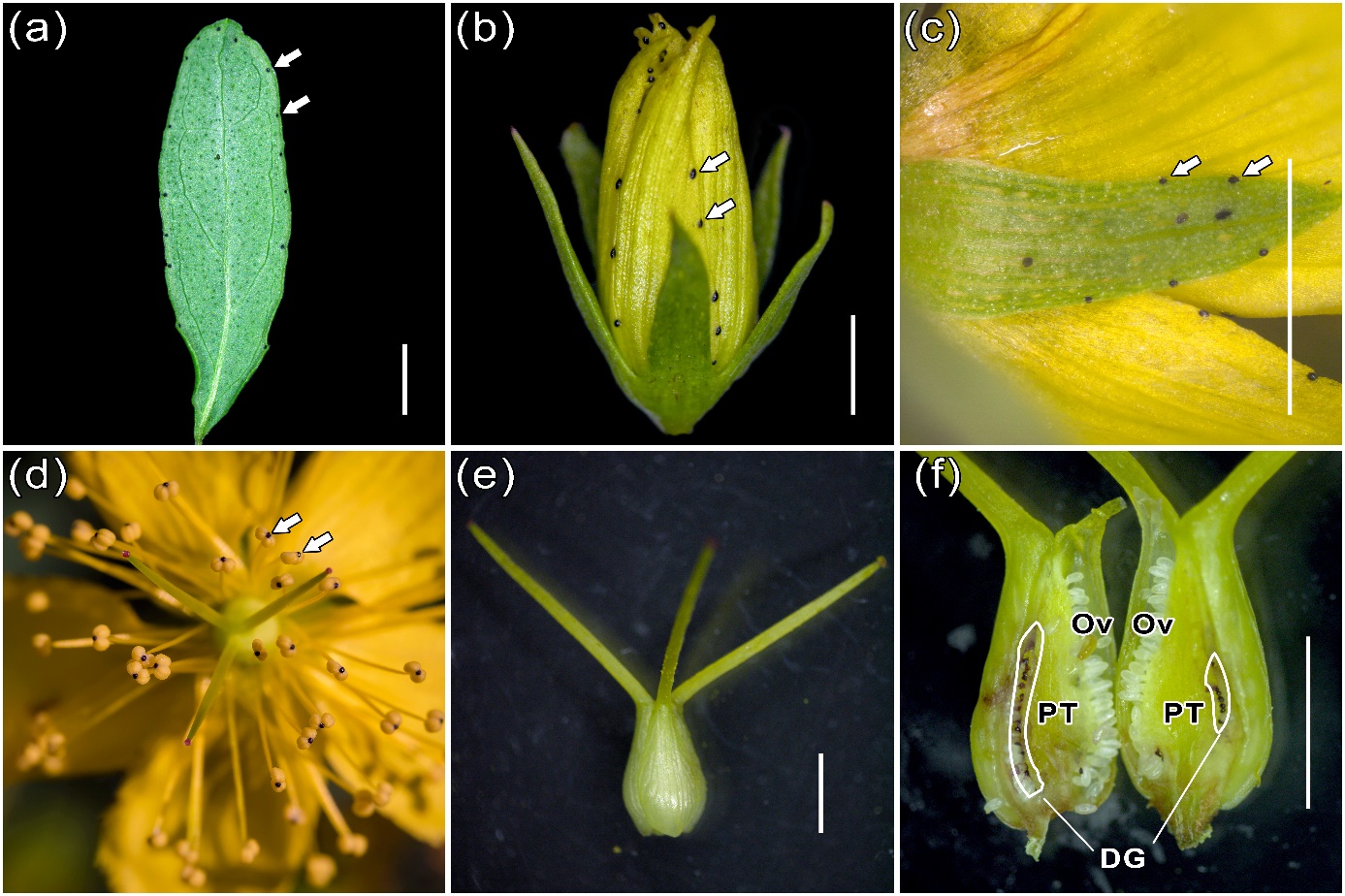


Figure S1 - Dark glands (containing hypericin) in different organs of H. perforatum. (**a**): Leaf rim; (**b**): Petals rim; (**c**): Sepal rim; (**d**): Anthers; (**e**): Pistil lateral view (no dark glands visible externally); (**f**): pistil longitudinal section (dark glands visible around the placental tissue). White arrows highlight the dark glands. Scale bars = 2mm; Ov = ovules; PT = placental tissue; DG = dark glands.


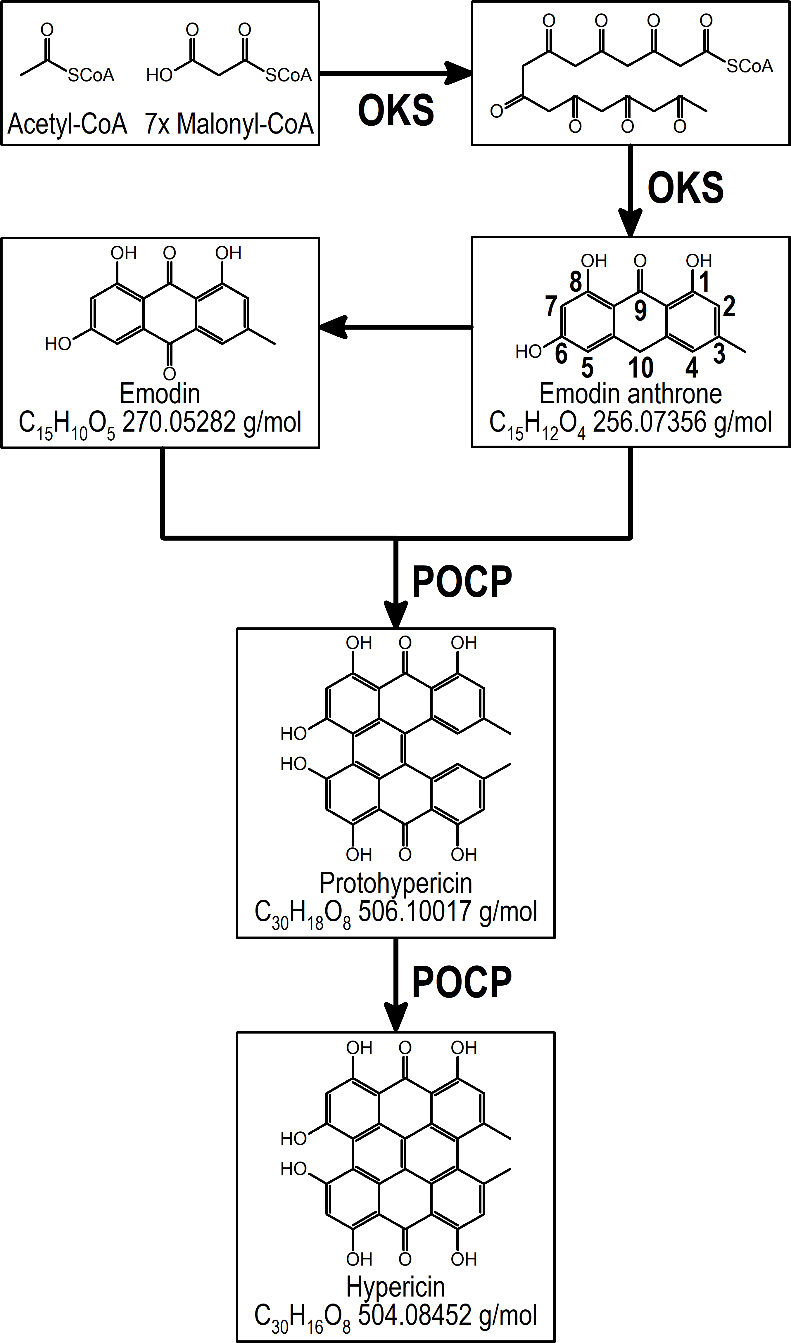


Figure S2 – Currently proposed hypericin biosynthesis pathway in Hypericum. Modified from Soták, et al., 2016a


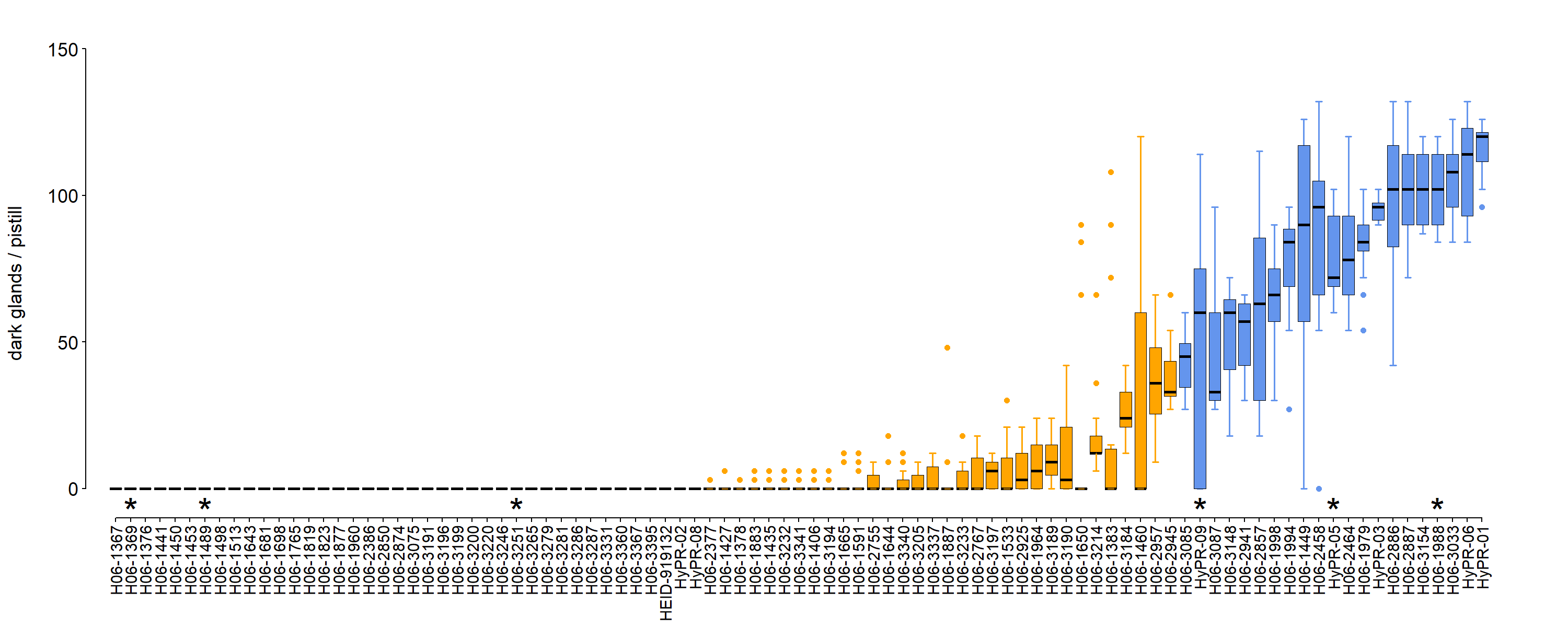


Figure S3 – Boxplots showing the observed distribution of dark glands per pistil for all 93 H. perforatum genotypes. The number of dark glands per pistil were obtained from five plants of each genotype from which three flowers per plant were investigated (n = 15). Heavily glanded G++ genotypes are shown in blue, while intermediately to weakly glanded G+ genotypes are coloured in orange. In glandless G- genotypes no dark glands were observed. Therefore, they appear as black lines (dark glands / pistil = 0). * indicates genotypes used for the transcriptome analyses of G- and G++ placental tissues.


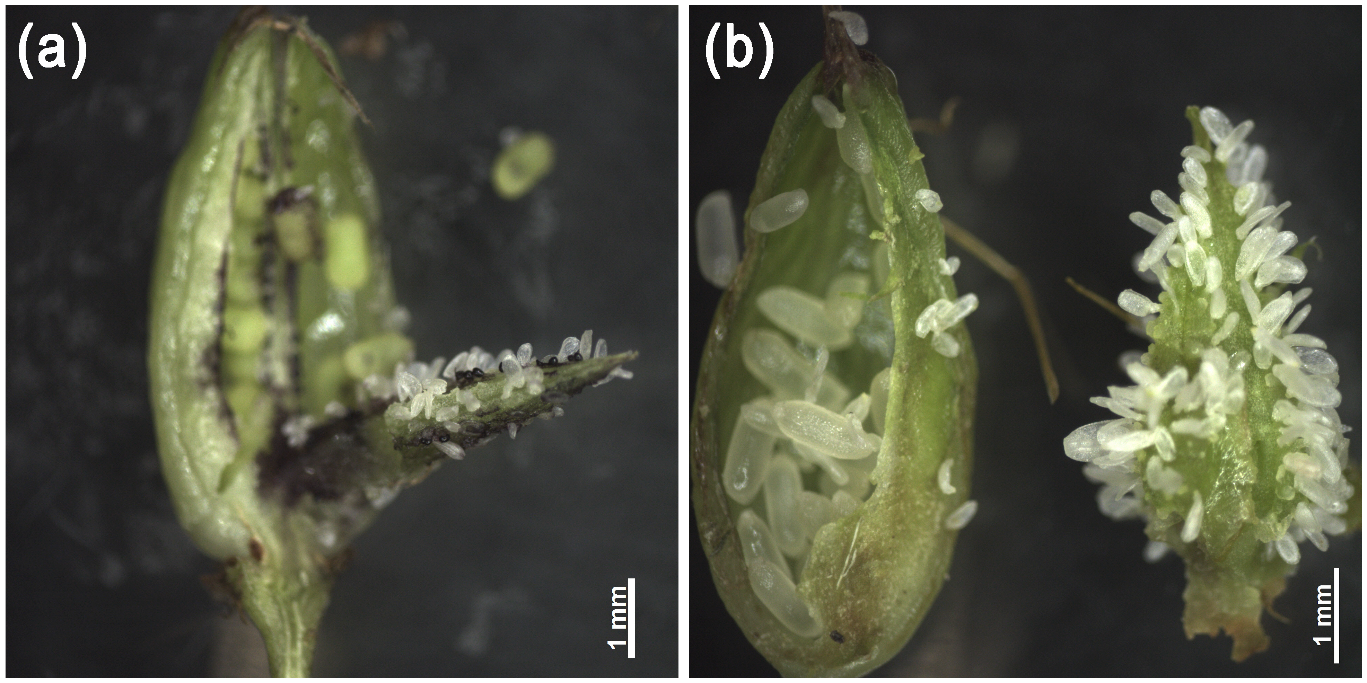


Figure S4 - Dissected capsules 14 days after open flower stage. (**a**): line H06-1194 characterized by G++ PT phenotype; (**b**): line H06-3251 characterized by a G- PT, no dark glands differentiation detected in the placenta even at later stages.


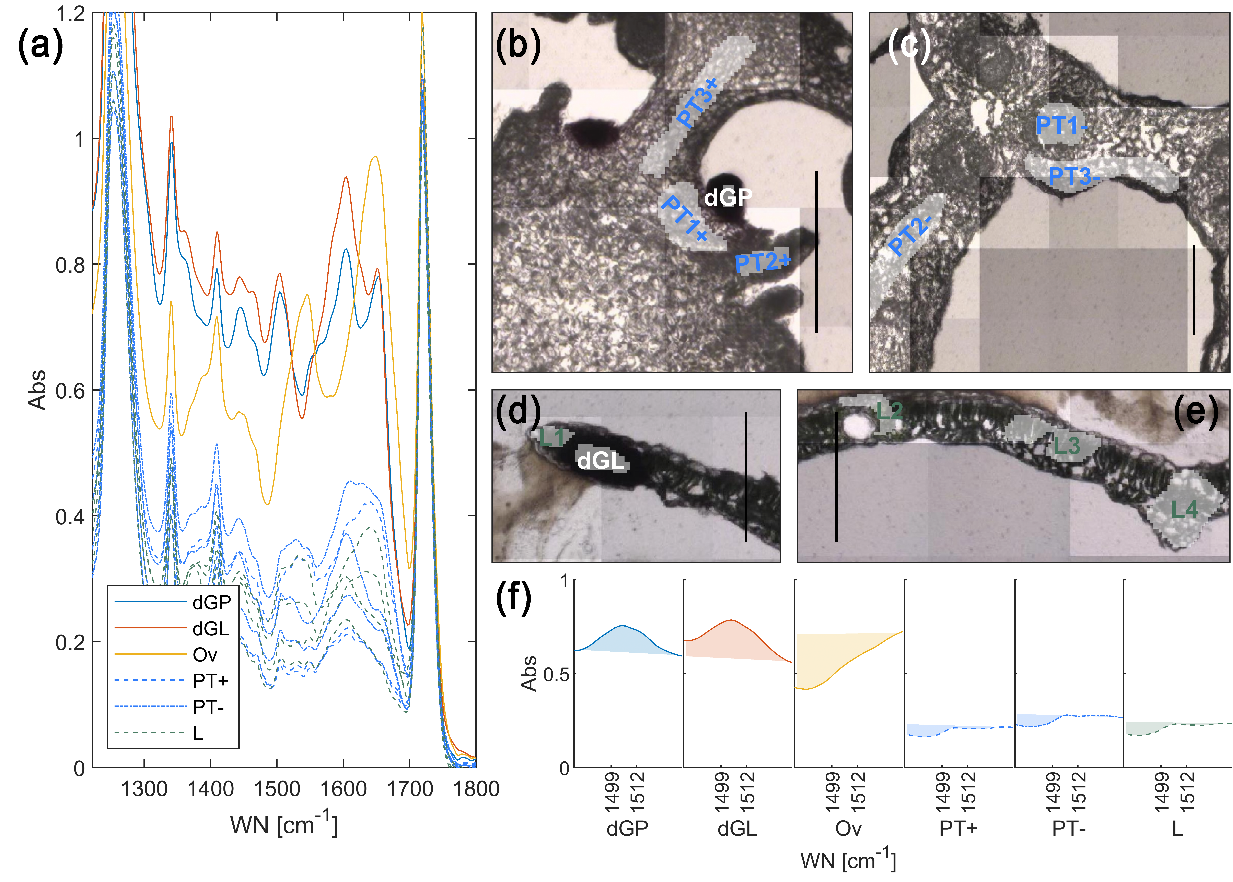


Figure S5 - Spectral analysis of different placental tissue and leave regions of H. perforatum by FTIR microspectroscopy. (**a**): Average spectra of regions of glanded (**b**) and glandless pistils (**c**) and Hypericum leaf tissue (**d**, **e**). (**f**): Integration of the spectral interval associated with the distinct dark gland fingerprint show only positive area values for dark gland tissue. dGP: pistil dark Gland, dGL: leaf dark Gland, Ov: ovule, PT+: placental tissue of glanded pistil, PT-: placental tissue of glandless pistil, L: leaf, Abs: absorbance, WN: wavenumber, Scale bar = 250 µm


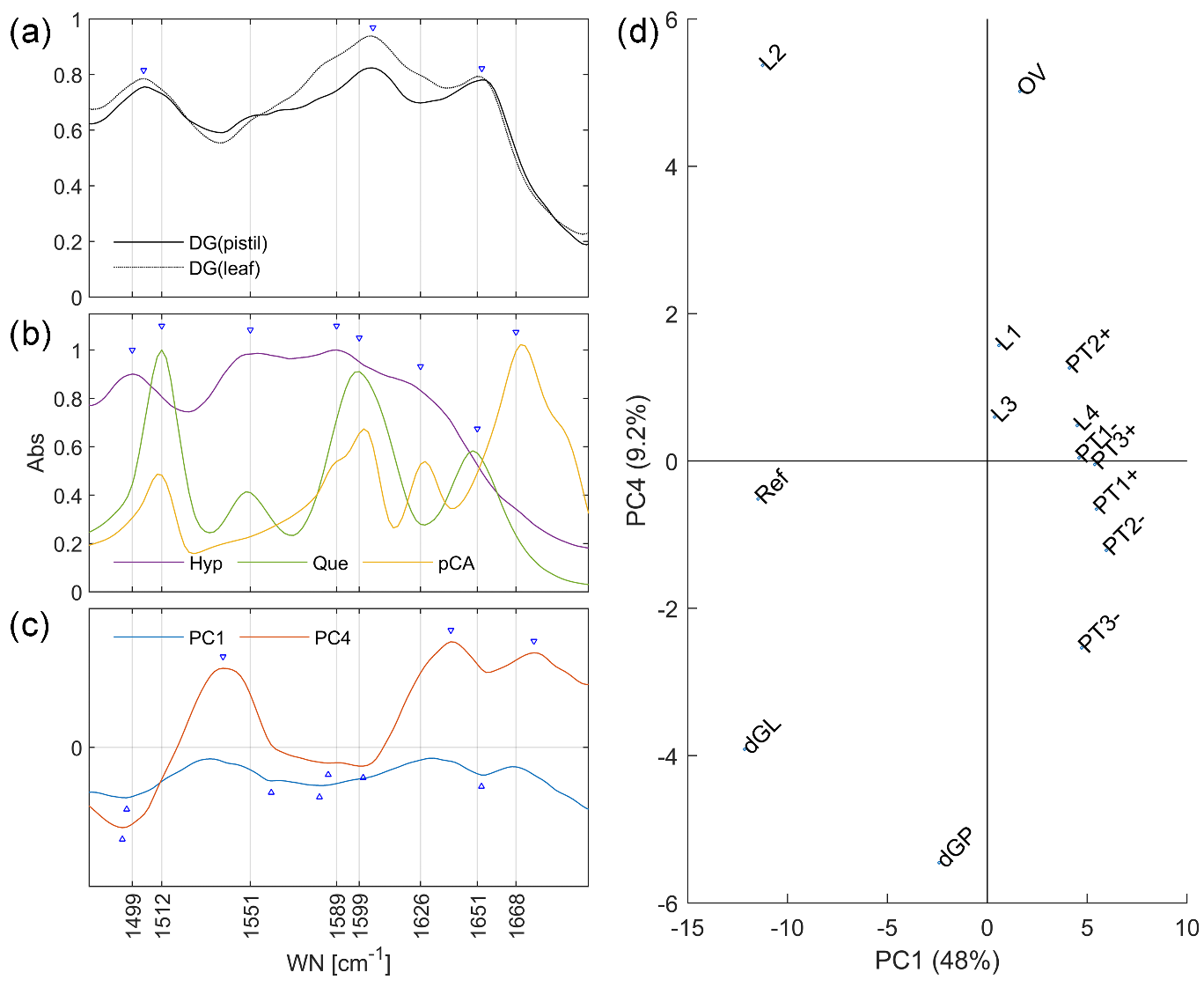


Figure S6 – Principle component analysis of H. perforatum infrared spectra. (**a**) Dark gland fingerprint shows a coherent fingerprint both in leaf and pistil tissue. (**b**) Raw spectra of basic components hypericin (Hyp), quercetin (Que) and p-coumaric acid (pCA) associated with dark glands by UHPLC-ESI-HRMS. (**c**) loading spectra of principle components 1 and 4 demonstrate spectral bands related to hypericin at 1499, 1551 and 1589 cm-1 and bands at 1512 (shoulder), around 1600 and 1651 cm^-1^ explaining quercetin like features are positively correlated with dark gland features (**d**) Principle component 1 and 4 score plot allow a differentiation between dark glands and other tissue regions of leaf and pistil.. Positively weighted features at 1535 and 1635 relate to the absence of Amide I and II bands within the gland tissue. dGP: pistil dark gland, dGL: leaf dark gland, Ov: ovule, PT+: placental tissue of glanded pistil, PT-: placental tissue of glandless pistil, L: leaf, Ref: FTIR substrate standard, Abs: absorbance, WN: wavenumber.


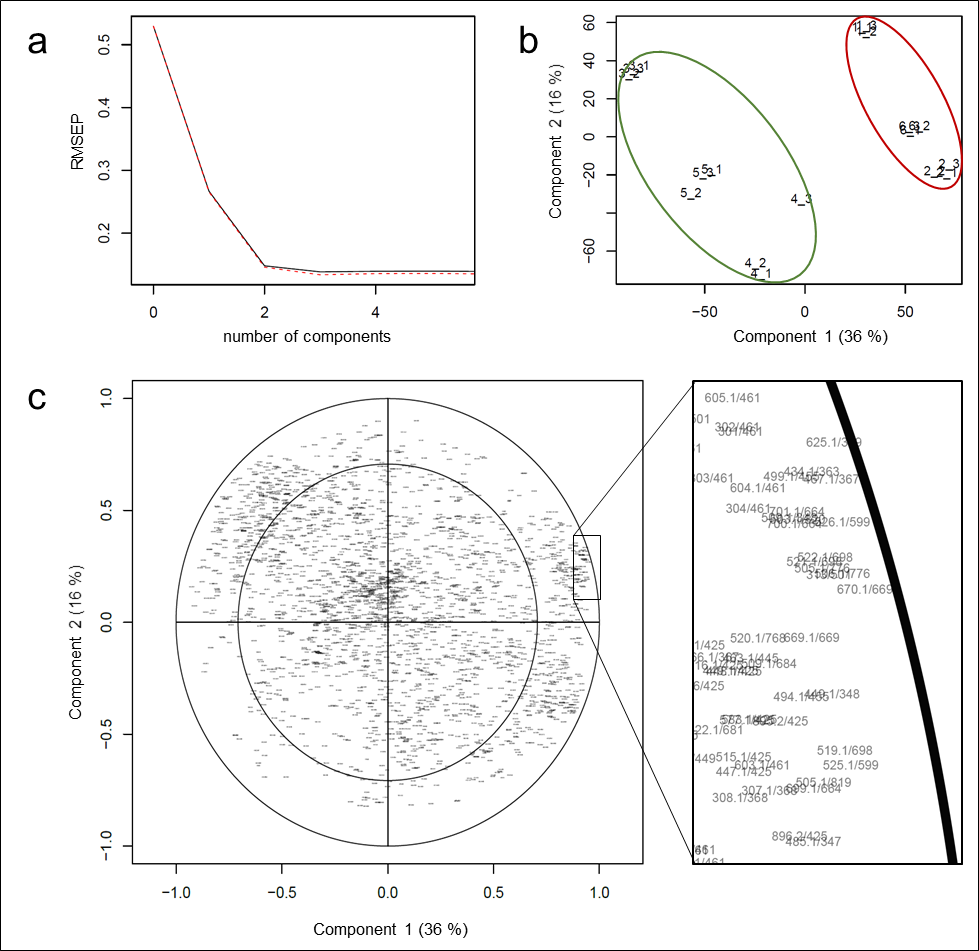


Figure S7 - PLS analysis of glanded versus glandless Hypericum pistils analyzed by UHPLC-ESI-HRMS in negative mode. (**a**): root mean squared error of prediction curve (black line = cross validation estimate, red dotted line = bias corrected cross validation estimate); (**b**): score plot (G++PT accessions (red): 1 = H06-1988, 2 = HyPR-03, 6 = HyPR-01; G- PT accessions (green): 3 = H06-1489, 4 = H06-1369, 5 = H06-3220); (**c**): correlation loading plot.


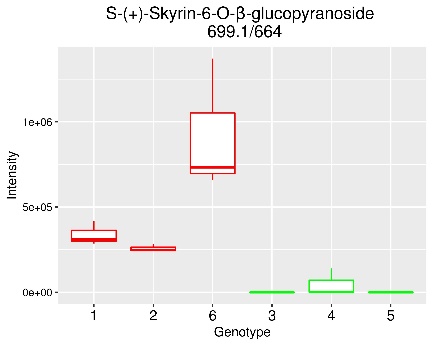

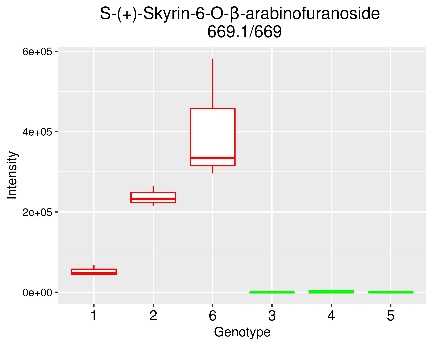

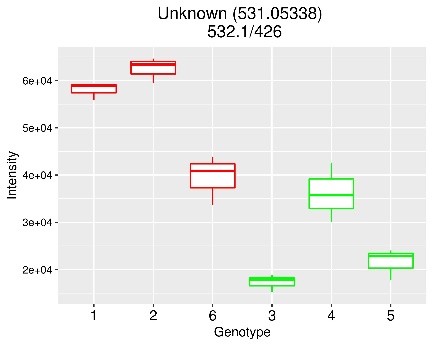

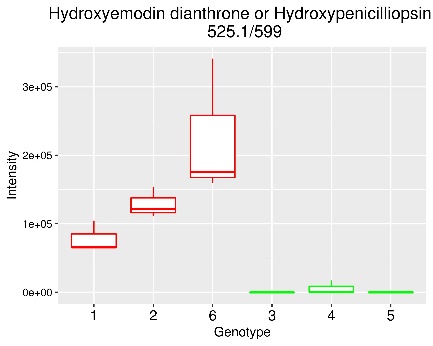

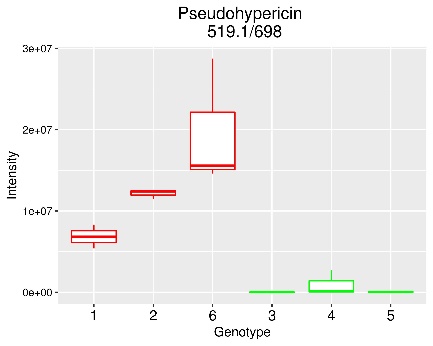

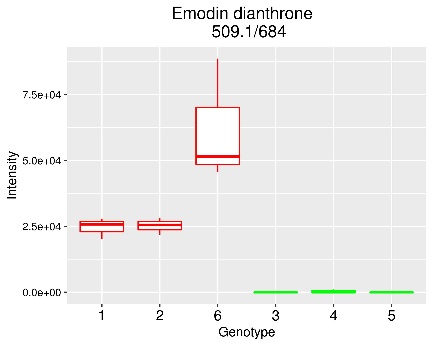

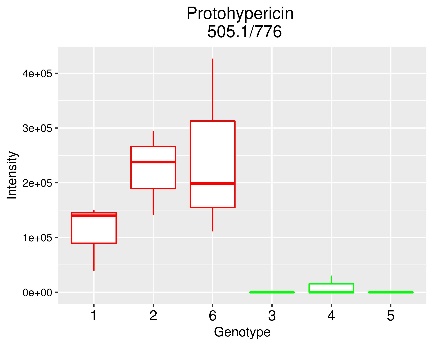

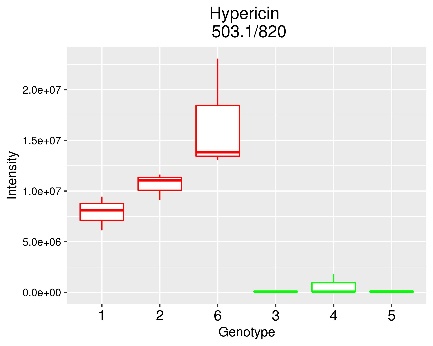

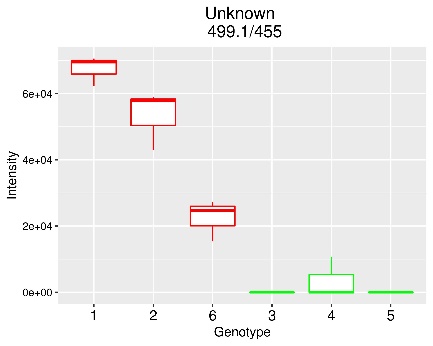

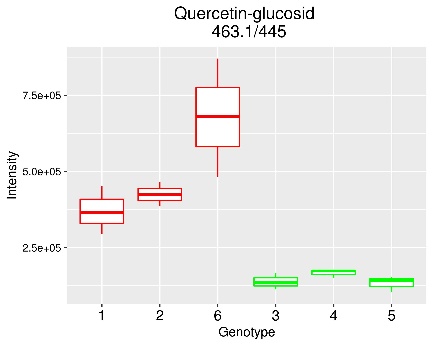

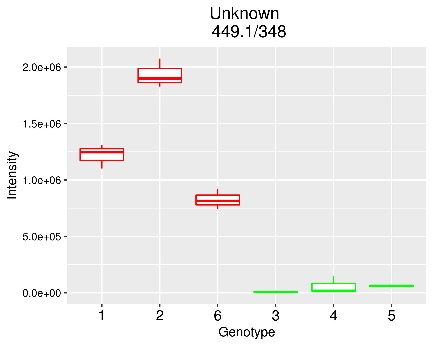

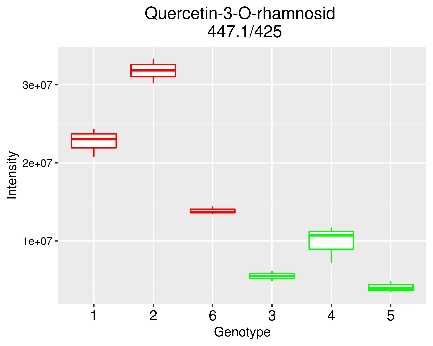

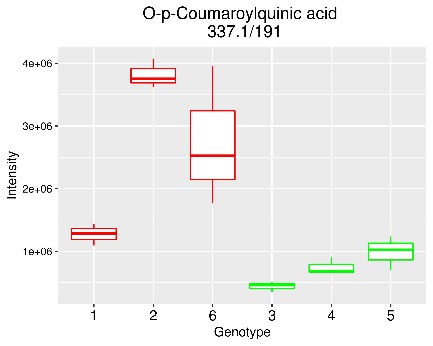

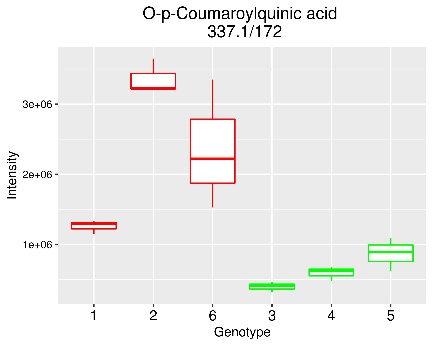

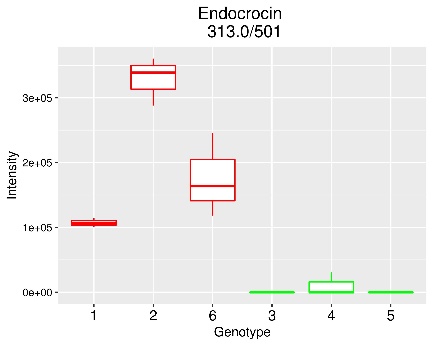

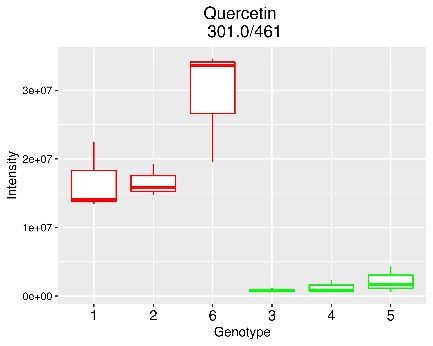

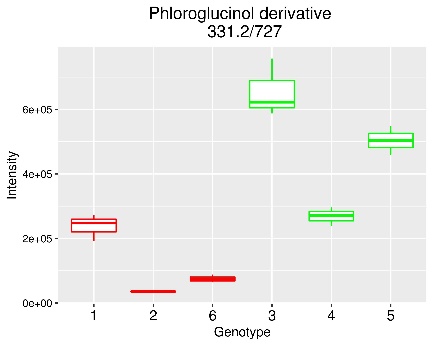

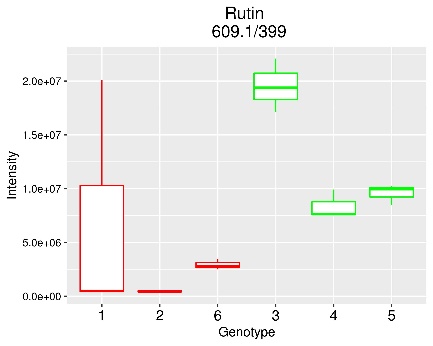


**18**

**17**

**16**

**13**

**15**

**14**

**12**

**11**

**10**

**9**

**8**

**7**

**3**

**6**

**5**

**4**

**2**

**1**

Figure S8 - MS intensity boxplots of selected correlating features **1**-**18** (Table 1). G++PT accessions (red): 1 = H06-1988, 2 = HyPR-03, 6 = HyPR-01; G- PT accessions (green): 3 = H06-1489, 4 = H06-1369, 5 = H06-3220. Each boxplot is based on three biological replicates each composed by 10 pistils of the same genotype.


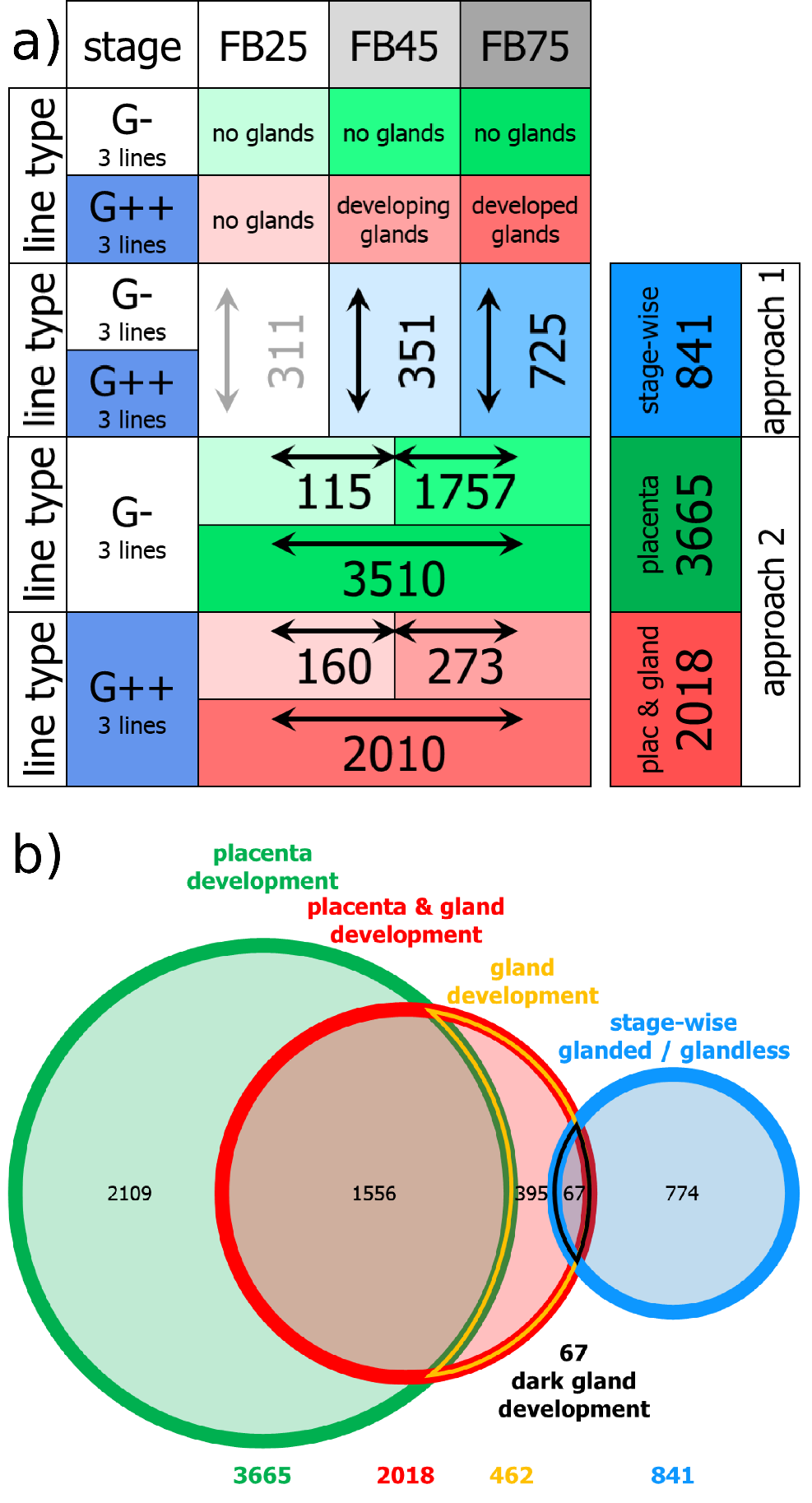


Figure S9 - Scheme of comparison approaches and Venn Diagram of DEGs (Differentially expressed genes) obtained with multiple comparison approaches. (**a**): vertical double headed arrows represent comparisons across line types and within stages. Horizontal arrows represent comparisons across stages and within types. (**b**): In Blue: 841 DEGs obtained with a stage wise approach comparison across G- PT and G++ PT. In green: 3665 DEGs from comparison across developmental stages within the G- PT lines. In red: 2018 DEGs from comparison across developmental stages within the G++ PT lines. In yellow: DEGs exclusively detected in the G++ PT lines. In black: 67 DEGs in common between the DEGs list of the stage-wise approach and the G++ PT specific DEGs of the type-wise approach.


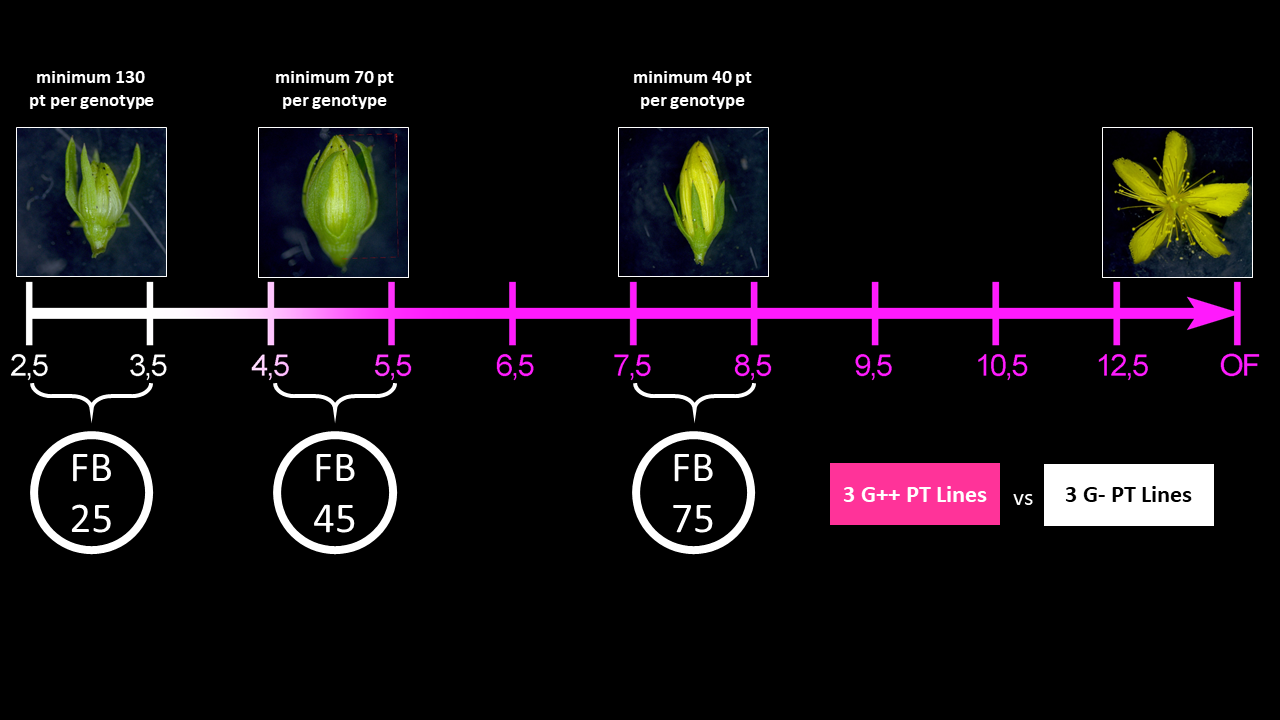


Figure S10 - Transcriptomics experimental design. **FB25** = placental tissues from flower buds 2.5 to 3.5 mm long; **FB45** = placental tissues from flower buds 4.5 to 5.5 mm long; **FB75** = placental tissues from flower buds 7.5 to 8.5 mm long; **pt** = placental tissues; **G- PT** = Glandless placental tissues genotypes; **G++ PT** = Glanded placental tissues genotypes; Numbers on the developmental scale refer to the flower buds length expressed in mm; the colorimetric gradient refers to the pre-differentiation stages (solid white), differentiation stage (white turning violet), post-differentiation stages (solid violet) **OF** = open flower.


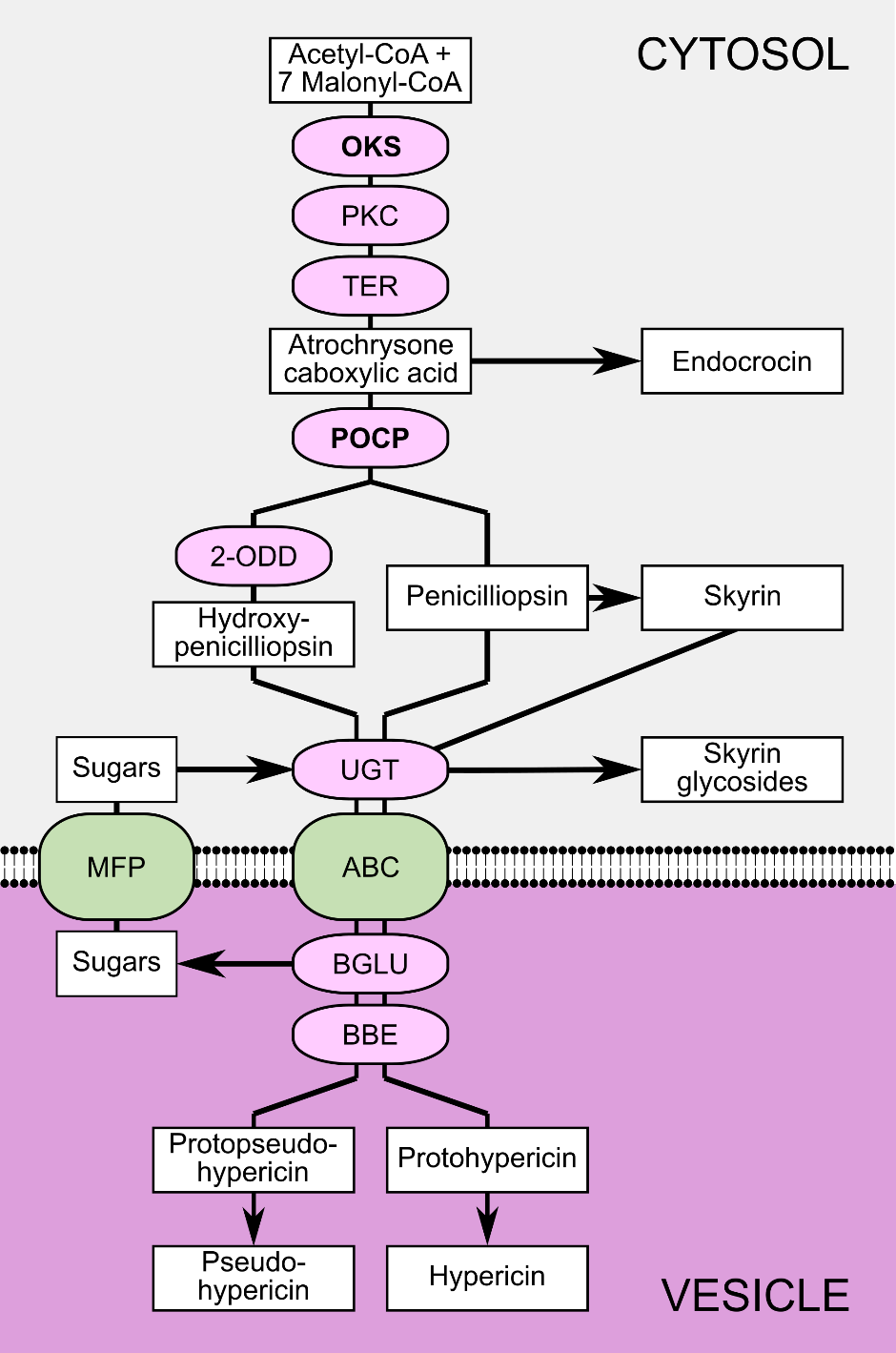


Figure S11 – Model of hypericin biosynthesis and transport based on our transcriptomics and metabolomics data. Round shapes represent proteins encoded by genes overexpressed in glanded placentas (pink = biosynthesis; green = transport). White rectangles represent metabolic compounds or precursors. OKS = Octaketide synthase; PKC = Polyketide cyclase; TER = Thioesterase; POCP = Phenolic oxidative coupling protein; 2-ODD = 2-oxoglutarate and Fe(II)-dependent dioxygenase; UGT = UDP-glucosyl transferase; MFP = Major facilitator protein; ABC = ABC transporter; BGLU = Beta glucosidase; BBE = Berberine bridge enzyme.
